# A pan-allelic human SIRPα-blocking antibody, ES004-B5, promotes tumor killing by enhancing macrophage phagocytosis and subsequently inducing an effective T-cell response

**DOI:** 10.1093/abt/tbae022

**Published:** 2024-08-28

**Authors:** Xiaofeng Niu, Chunnian Wang, Haixia Jiang, Rui Gao, Yefeng Lu, Xiaoli Guo, Hongping Zhou, Xue Cui, Jun Sun, Quan Qiu, Dawei Sun, Hongtao Lu

**Affiliations:** Elpiscience Biopharma, BLDG. 3, 998 Halei RD, Pudong, Shanghai 201203, P.R. China; Elpiscience Biopharma, BLDG. 3, 998 Halei RD, Pudong, Shanghai 201203, P.R. China; Elpiscience Biopharma, BLDG. 3, 998 Halei RD, Pudong, Shanghai 201203, P.R. China; Elpiscience Biopharma, BLDG. 3, 998 Halei RD, Pudong, Shanghai 201203, P.R. China; Elpiscience Biopharma, BLDG. 3, 998 Halei RD, Pudong, Shanghai 201203, P.R. China; Elpiscience Biopharma, BLDG. 3, 998 Halei RD, Pudong, Shanghai 201203, P.R. China; Elpiscience Biopharma, BLDG. 3, 998 Halei RD, Pudong, Shanghai 201203, P.R. China; Elpiscience Biopharma, BLDG. 3, 998 Halei RD, Pudong, Shanghai 201203, P.R. China; Elpiscience Biopharma, BLDG. 3, 998 Halei RD, Pudong, Shanghai 201203, P.R. China; Elpiscience Biopharma, BLDG. 3, 998 Halei RD, Pudong, Shanghai 201203, P.R. China; Elpiscience Biopharma, BLDG. 3, 998 Halei RD, Pudong, Shanghai 201203, P.R. China; Elpiscience Biopharma, BLDG. 3, 998 Halei RD, Pudong, Shanghai 201203, P.R. China

**Keywords:** cancer immunotherapy, CD47, SIRPα, macrophage phagocytosis

## Abstract

As a major immune cell type in the tumor microenvironment, tumor-associated macrophages secrete suppressive factors that can inhibit antitumor immunity and promote tumor progression. One approach trying to utilize macrophages for immunotherapy has been to block the CD47-SIRPα axis, which mediates inhibitory signaling, to promote phagocytosis of tumor cells. Many CD47-targeted agents, namely, anti-CD47 antibodies and SIRPα fusion proteins, were associated with a diverse spectrum of toxicities that limit their use in clinical settings. Universal expression of CD47 also leads to a severe “antigen sink” effect of CD47-targeted agents. Given that the CD47 receptor, SIRPα, has a more restricted expression profile and may have CD47-independent functions, targeting SIRPα is considered to have distinct advantages in improving clinical efficacy with a better safety profile. We have developed ES004-B5, a potentially best-in-class pan-allelic human SIRPα-blocking antibody using hybridoma technology. ES004-B5 binds to major human SIRPα variants through a unique epitope with high affinity. By blocking CD47-induced inhibitory “don't-eat-me” signaling, ES004-B5 exerts superior antitumor activity in combination with anti-tumor-associated antigen antibodies *in vitro* and *in vivo*. Unlike CD47-targeted agents, ES004-B5 exhibits an excellent safety profile in nonhuman primates. ES004-B5 has potential to be an important backbone for SIRPα-based combination therapy and/or bispecific antibodies, which will likely overcome the limitations of CD47-targeted agents encountered in clinical settings.

## Teaser

**Statement of Significance:** SIRPα-targeted therapy will likely overcome the limitations of anti-CD47 antibodies or SIRPα fusion proteins encountered in clinical settings. We have developed ES004-B5, a potentially best-in-class pan-allelic human SIRPα-blocking antibody, which has potential to be an important backbone of SIRPα-based combination therapies or bispecific antibodies.

## Introduction

Immune checkpoint (ICP) inhibitors targeting programmed cell death protein 1 (PD-1), programmed death-ligand 1 (PD-L1), cytotoxic T-lymphocyte-associated protein 4 (CTLA-4), and lymphocyte-activation gene 3 (LAG3) have revolutionized cancer treatment. However, despite the fact that these T-cell-based immunotherapies do provide safe and durable treatment responses, only a small subset of patients see long-term benefit. A key factor involved in initial resistance to ICP inhibitors is the lack of tumor T-cell infiltration, characterizing so-called “cold tumors” [1]. In contrast, tumor-associated macrophages (TAMs) are abundant in the tumor microenvironment (TME) of most solid tumors, accounting for 30%–50% of stromal cells [2]. As a major immune cell type in the TME, TAMs secrete suppressive factors that can inhibit antitumor immunity and promote tumor progression. Increased infiltration of macrophages is correlated with poor clinical prognosis in many cancers [3]. Hence, TAMs have emerged as an attractive target for cancer immunotherapy. One important approach being utilized in TAM-targeted immunotherapies is to block the CD47-SIRPα axis, which mediates inhibitory signaling, to promote TAM-mediated phagocytosis of tumor cells.

Signal regulatory protein alpha (SIRPα) contains three Ig-like domains, a single transmembrane region, and a cytoplasmic region that contains four tyrosine residues with immunoreceptor tyrosine–based inhibition motifs (ITIMs) [4]. SIRPα is expressed mainly on myeloid cells and to varying levels on neurons [5, 6]. The functional ligand of SIRPα was identified as CD47 in rodents and human [7, 8] and is also known as integrin-associated protein (IAP) because it is associated with integrins such as α_v_β_3_ [9]. CD47 is expressed on virtually all cells including erythrocytes and platelets, which has a physiological role in self-recognition [10]. Ligation of CD47 to SIRPα on macrophages delivers a “don't-eat-me” signaling to suppress phagocytosis [11]. Upon being engaged by CD47, the phosphorylation of the ITIMs in the SIRPα cytoplasmic tail is promoted, primarily by Src family kinases [12]. The phosphorylated SIRPα ITIMs subsequently recruit and activate Src homology domain 2-containing phosphatases-1 (SHP-1) and -2 (SHP-2) [4, 13, 14] and thereby block phagocytosis, potentially by preventing myosin-IIA accumulation at phagocytic synapses [15]. Loss of CD47-SIRPα inhibitory signaling in macrophages results in more rapid clearance of circulating platelets and erythrocytes [16, 17]. Tumor cells frequently overexpress CD47 to evade macrophage-mediated destruction [18], and high CD47 expression in a variety of tumors predicts poor prognosis [18–21]. Numerous studies have demonstrated that blocking CD47/SIRPα “don't-eat-me” signaling with anti-CD47 monoclonal antibody (mAb), SIRPα fusion protein, or anti-SIRPα mAb stimulates macrophage phagocytosis of tumor cells, which eventually triggers adaptive immune response to immunogenic tumor cells and inhibition of tumor growth in mouse tumor models [22–26]. Currently, a number of agents targeting CD47 have proceeded to clinical trials. Despite the fact that some of them have demonstrated early clinical responses in hematologic cancers in combination with chemotherapy or antibodies targeting tumor-associated antigens (TAAs) [26–29], they have been reported to cause dose-limiting toxicities including anemia and thrombocytopenia [30–34]. In addition, universal expression of CD47 necessitates high doses of CD47-targeted agents to overcome peripheral “antigen sink” issues and engage sufficient target in tumors, which, in turn, may increase the risk of causing treatment-related side effects. Given that the CD47 receptor, SIRPα, has a more restricted expression profile, targeting SIRPα is considered to have distinct advantages over CD47 targeting in improving clinical efficacy with a better safety profile.

Currently, multiple drug candidates targeting SIRPα for cancer immunotherapy are being in clinical development. In phase 1 clinical studies, the first-in-class anti-SIRPα antibody BI 765063 (OSE-172, Boehringer Ingelheim) was well tolerated with no unexpected toxicities and demonstrated dose-related evidence of target engagement and antitumor activity as monotherapy and in combination with anti-PD-1 treatment in solid tumors [35, 36]. However, SIRPα is highly polymorphic and BI 765063 is a huSIRPα V1-specific antibody, suggesting that BI 765063 may not be effective in patients with other *SIRPA* alleles. In addition to SIRPα, the SIRP family contains other members such as signal regulatory protein beta (SIRPβ, also known as SIRP beta1) and signal regulatory protein gamma (SIRPγ, also known as SIRP beta2), whose extracellular domain (ECDs) are highly homologous to that of SIRPα (84.8% for SIRPβ and 80.1% for SIRPγ) [37]. The expression profile of SIRPβ is similar to that of SIRPα. However, SIRPγ is highly expressed on T cells but not myeloid cells. It was reported that adhesion of human T cells to antigen-presenting cells via SIRPγ-CD47 interaction co-stimulates T-cell proliferation [38]. Thus, successful therapeutic targeting of SIRPα in diverse patient populations necessitates a pan-allelic anti-SIRPα antibody that cross reacts with pan-SIRPα alleles but does not interfere with T-cell functions.

In this study, we report that we have developed ES004-B5, a potentially best-in-class pan-allelic human SIRPα-blocking antibody, as a drug candidate for the treatment of cancer patients. ES004-B5 binds to major human SIRPα variants with high affinity. It recognizes a unique epitope on SIRPα that is distinct from known competitor molecules. ES004-B5 potently blocks CD47-SIRPα interaction. Through blocking CD47-induced inhibitory “don't-eat-me” signaling, ES004-B5 effectively promoted macrophage phagocytosis of tumor cells and potentiated tumor cells opsonization by anti-TAA antibodies. In mouse syngeneic tumor models, ES004-B5 not only significantly inhibited tumor growth *in vivo* as a single agent but also greatly enhanced the antitumor activity of anti-TAA antibodies. Although ES004-B5 binds to SIRPγ expressed on T cells, it does not negatively impact T-cell activation. CD8 T cells and IFNγ are essential for the antitumor effect of ES004-B5/anti-TAA combination therapy. Unlike CD47-targeted agents, ES004-B5 exhibits excellent safety profile in nonhuman primates (NHPs).

## Materials and methods

### Antibodies and reagents

ES004-B5 was generated using hybridoma technology. SJL/J mice were immunized with gold microparticles (Bio-Rad) coated with a human SIRPα (huSIRPα) variant V1 mammalian expression plasmid. The immunized mice with highest titers were sacrificed to collect spleen cells for fusion with Sp2/0 myeloma cells to generate hybridomas. SIRPα-reactive antibodies were screened by binding to SIRPα recombinant proteins or SIRPα-expressing cells and anti-SIRPα functional antibodies were identified by blocking assay and phagocytosis assay. One lead antibody (ES004-B5 precursor) was humanized by complementarity-determining region (CDR) grafting onto a human IgG4 κ backbone containing S228P mutation to create ES004-B5. ES0060028 was also generated using hybridoma technology by first immunizing BALB/c mice with gold microparticles (Bio-Rad) coated with a human Claudin 18.2 (huCLDN18.2) mammalian expression plasmid. The immunized mice with highest titers were sacrificed to collect spleen cells for fusion with Sp2/0 myeloma cells to generate hybridomas. CLDN18.2-reactive antibodies were screened by enzyme-linked immunosorbent assay (ELISA) for binding to recombinant CLDN18.2 protein or by flow cytometry for binding to CLDN18.2-expressing cells, and functional antibodies then selected by antibody-dependent cellular cytotoxicity assay. One lead antibody was humanized by CDR grafting onto a human IgG1 κ backbone to create ES0060028.

ESD05_2719 was generated using a yeast display approach. Female alpacas were immunized with human PD-L1 recombinant protein (Sino Biological). A variable domain of heavy-chain antibody (VHH) yeast library with a total diversity of 1.5 × 10^9^ was constructed using RNA extracted from peripheral blood mononuclear cells (PBMCs) of immunized alpacas. PD-L1 binders were enriched through two rounds of magnetic activated cell sorting (MACS) and three rounds of fluorescence-activated cell sorting (FACS). In the last round of FACS, a high affinity PD-1 variant was introduced for the PD-L1 blocker screening [39]. PD-L1 blockers (VHHs) were sequenced and fused to a human IgG1 Fc containing C220A mutation. The lead anti-PD-L1 specific functional antibody ESD05_2719 was identified by detecting B7 family protein-binding selectivity, reporter assay, and mixed lymphocyte reaction (MLR).

Two human IgG4 anti-SIRPα reference antibodies, HEFLB (an analogue of BI 765063) and hu1H9 (an analogue of FSI-189, Gilead), were produced at Biointron according to patents WO 2017/178653 A2 (SEQ ID NO: 42 and 45) and WO 2019/023347 Al (SEQ ID NO: 7 and 8), respectively. Anti-PD-L1 single-domain antibody KN035 was produced at Biointron according to patent CN 111116747 A (SEQ ID NO: 33). Anti-SIRPγ antibody LSB2.20 was purchased from Biolegend. Human IgG1, human IgG4, and mouse IgG1 isotype control antibodies were all purchased from Biointron as were the following recombinant proteins: mouse Fc-tagged huCD47 ECD, mouse Fc-tagged huSIRPα V1 ECD, and 6 × His tagged huSIRPα V8 ECD. Recombinant 6 × His tagged huSIRPα V2 ECD, huSIRPγ ECD, and cynomolgus SIRPα ECD proteins were purchased from KACTUS. 6 × His tagged huSIRPα V1 ECD recombinant protein was purchased from Sino Biological.

### Cell lines and cell culture

CHOK1/hSIRPα V1 and CHOK1/cSIRPα cell lines were purchased from Chempartner. The CHOK1/hSIRPα V2 cell line was purchased from Kyinno. These three cell lines were cultured in F-12K medium (Gibco) containing 10% fetal bovine serum (FBS, Gibco) and 6 μg/ml puromycin (Gibco). DLD-1, Jurkat, and Raji cell lines were purchased from the American Type Culture Collection (ATCC) and cultured in RPMI 1640 medium (Gibco) containing 10% FBS (Gibco). The HCT-116 cell line was purchased from ATCC and cultured in MoCoy’s 5A medium (ATCC) containing 10% FBS (Gibco). The Raji-hPD-L1 cell line was purchased from InvivoGen and cultured in Iscove's Modified Dulbecco's Medium (IMDM, Gibco) containing 10% FBS (Gibco) and 10 μg/ml blasticidin (InvivoGen). The K562/SHP-1^+^hSIRPα^+^CD47^−^ cell line was generated by Elpiscience and cultured in RPMI 1640 medium (Gibco) containing 10% FBS (Gibco), 100 μg/ml hygromycin (Gibco), and 6 μg/ml puromycin (Gibco). Briefly, full-length huSIRPα V1 was engineered with a small β-galactosidase fragment (ED) fused to its C-terminal, and the SH2-domain of SHP-1 was engineered with the complementing β-galactosidase fragment. These constructs were stably expressed in human CD47 knockout K562 cells to generate K562/SHP-1^+^hSIRPα^+^ CD47^−^ cells. All above cell lines were culture at 37°C in 5% CO_2_.

### Primary cell isolation and differentiation

Human PBMCs isolated from blood of healthy donors were purchased from SailyBio or MILESTONE BIO. CD14^+^ monocytes were enriched using Human CD14 MicroBeads (Miltenyi). T cells were enriched using EasySep™ Human T Cell Isolation Kit (Stemcell). Human monocyte–derived macrophages (hMDMs) were generated from PBMCs cultured in RPMI 1640 medium (Gibco) containing 10% FBS (Gibco), 50 ng/ml human macrophage colony-stimulating factor (M-CSF, R&D Systems) for 7 days at 37°C in 5% CO_2_. For human monocyte-derived dendritic cells (MoDCs) generation, CD14^+^ monocytes enriched from PBMCs of healthy donors were cultured in RPMI 1640 medium (Gibco) containing 10% fetal bovine serum (Gibco), 50 ng/ml human granulocyte-macrophage colony-stimulating factor (GM-CSF, R&D Systems), and 25 ng/ml human IL-4 (R&D Systems) for 5 days at 37°C in 5% CO_2_ and then stimulated with 1 μg/ml LPS (Sigma-Aldrich) for additional 24 h for maturation.

### Surface plasmon resonance

The binding affinities and kinetics of anti-SIRPα mAbs to huSIRPα variants V1, V2, V8, cynomolgus SIRPα, and huSIRPγ were determined using surface plasmon resonance (SPR) technique on a Biacore T200 instrument (Cytiva) at 25°C. First, according to the manual of the human antibody capture kit (Cytiva), antihuman IgG (Fc) antibody was immobilized onto CM5 sensor chips (Cytiva) to generate the capture surfaces for the antibodies to be tested. About 7000 RU of anti-human IgG (Fc) antibody per flow cell was achieved. Then, each antibody was detected using the same method as follows: antibody in 1× HBS-EP+ running buffer (0.5 μg/ml) was injected at a flow rate of 10 μl/min to achieve 80 ± 12 RU of captured antibody per sample flow cell, simultaneously a buffer blank was injected at the same rate through reference flow cell; 6 × His tagged-SIRP analyte (30–0.9375 nM in running buffer, 2-fold serial dilution) or a buffer blank was injected through both sample flow cell and reference flow cell for 150–180 s at 30 μl/min, followed by a dissociation cycle of 180 s; chip surface was then washed with running buffer and regenerated with regeneration buffer (Glycine1.5) for 30 s at 30 μl/min. Biosensor data were double-referenced by subtracting the interspot data (the response of SIRP analyte in reference flow cell) from the reaction spot data (the response of SIRP analyte in sample flow cell) and then subtracting the response of the buffer blank analyte. Using global analysis, association–dissociation curves for the interactions of the test antibodies with the analytes were generated and fitted with the 1:1 binding model using Biacore T200 analysis software V10 to calculate maximum response (Rmax), chi-square (Chi^2^), association rate constants (ka), and dissociation rate constants (kd). Equilibrium dissociation constants (KD) were then obtained by calculating the ratios of kd/ka.

### SIRPα binding and blocking assays

For binding ELISA, serial dilutions of the test antibodies were incubated in a 6× His tagged huSIRPα V8 ECD recombinant protein–coated 96-well ELISA plate for 1 h at room temperature (RT). After unbound antibodies were removed by washing with PBS/0.05% Tween 20 (PBST), HRP-conjugated goat antihuman IgG Fcγ fragment–specific secondary antibody (Jackson ImmunoResearch) was added and incubated for 1 h at RT. After washing with PBST, color development was conducted by adding 3,3′5,5′-tetramethylbenzidine (TMB) solution (Biopanda) and reaction was stopped with 1 M HCl. Absorbance at 450 nM (OD450) and 570 nM (OD570) was then measured using a Versa Max (Molecular Devices).

For FACS-based binding assays, serial dilutions of the test antibodies were incubated with huSIRPα V1-overexpressing CHOK1/hSIRPα V1 cells, huSIRPα V2-overexpressing CHOK1/hSIRPα V2 cells, or cynomolgus SIRPα-overexpressing CHOK1/cSIRPα cells for 1 h at 4°C. After unbound antibodies were removed by washing with FACS buffer (DPBS containing 2% FBS), Alexa Fluor 647 conjugated goat anti-human IgG Fcγ fragment–specific secondary antibody (Jackson ImmunoResearch) was added and incubated with the cells for 30 min at 4°C. After washing with FACS buffer, cells were then analyzed by a flow cytometer (FACSCanto II, BD Biosciences).

The CD47/SIRPα interaction blocking activity of anti-SIRPα mAbs was assessed by FACS-based competition assays. Mouse Fc-tagged huCD47 ECD recombinant protein was incubated with CHOK1/hSIRPα V1 or CHOK1/hSIRPα V2 cells in the presence of serial dilutions of the test antibodies for 1 h at 4°C. After unbound huCD47 recombinant protein was removed by washing with FACS buffer, Alexa Fluor 647 conjugated goat anti-mouse IgG Fcγ fragment specific secondary antibody (Jackson ImmunoResearch) was added and incubated with the cells for 1 h at 4°C. After washing with FACS buffer, cells were then analyzed by a flow cytometer (FACSCanto II, BD Biosciences). The blocking activity of each sample was determined by quantitating the blockade of huCD47 recombinant protein binding to CHOK1/hSIRPα V1 or CHOK1/hSIRPα V2 cells.

### SIRPα/SHP-1 recruitment assay

SIRPα/SHP-1 recruitment assay was developed to assess the activity of anti-SIRPα mAbs to neutralize CD47-induced SIRPα signaling. K562/SHP-1^+^hSIRPα^+^CD47^−^ cells and Jurkat cells were seeded into 96-well plates at a ratio of 1:4. Serial dilutions of the test antibodies were incubated with the cells overnight at 37°C. On the second day, Gal-Screen Reaction Buffer (Applied Biosystems) was added and incubated with the cells for around 60 min at 28°C. Chemiluminescence was then detected using an Infinite F Plex (TECAN) as a measure of reporter activity for CD47-induced SIRPα signaling. The blocking activity of each sample was determined by quantitating the blockade of β-galactosidase activity.

### Epitope binning

Anti-SIRPα mAbs were tested in an ELISA-based competition assay to cluster antibodies with similar binding epitopes. A 96-well ELISA plate was coated with 1 μg/ml of the test antibodies overnight at 4°C. The plate was blocked with blocking buffer (2% BSA in PBST) for 2 h at 37°C. After 15 min of preincubation at RT, the mixture of 20 μg/ml of soluble form test antibodies and 1.2 nM of mouse Fc-tagged huSIRPα V1 ECD recombinant protein was added to the test antibodies-coated plate and incubated for 1 h at 37°C. After unbound proteins were removed by washing with PBST, HRP conjugated goat anti-mouse IgG Fc fragment specific secondary antibody (Sigma-Aldrich) was added and incubated for 1 h at 37°C. After washing with PBST, color development was conducted by adding TMB solution (Biopanda) and reaction was stopped with 1 M HCl. OD450 was then measured using a Versa Max (Molecular Devices). Competition ratios were determined by quantitating the blockade of huSIRPα V1 recombinant protein binding to the test antibodies coated on the ELISA plate.

### Hydrogen deuterium exchange mass spectrometry

Epitope mapping using hydrogen deuterium exchange mass spectrometry (HDX-MS) was conducted at Shanghai Institute of Materia Medica. 5 µM ES004-B5 precursor or HEFLB was pre-incubated with or without huSIRPα V1 ECD recombinant protein at a molar ratio of 1:2 for 30 min before hydrogen deuterium exchange reaction. Then, 4 μl of antibody/antigen complex was diluted with 16 μl D_2_O exchange buffer (50 mM HEPES, pH 7.4, 50 mM NaCl, 2 mM DTT in D_2_O) and incubated for 60 s at 4°C and quenched by adding 20 μl ice-cold quenching buffer consists of 100 mM NaH_2_PO_4_, 1 M tris (2-carboxylethyl) phosphine (TCEP). Each quenched sample was immediately injected into the LEAP Pal 3.0 HDX platform. Proteins were digested online in an immobilized pepsin column (2 mm × 2 cm) at a speed of 120 μl/min, and then, the digested peptides were captured and desalted on a C18 PepMap300 trap column (ThermoFisher). Peptides separation was achieved with a 2.1 mm × 5 cm C18 separating column (1.9 μm Hypersil Gold, ThermoFisher) with a 6 min linear gradient of 4%–40% CH_3_CN and 0.3% formic acid. All these procedures were conducted at 4°C. Mass spectrometric data were acquired using a Fusion Orbitrap mass spectrometer (ThermoFisher) with a measured resolving power of 65 000 at *m*/*z* of 400. HDX analyses were performed in triplicate with one preparation of each protein/ligand complex. The intensity weighted mean *m*/*z* centroid value of each peptide envelope was calculated and subsequently converted into a percentage of deuterium incorporation. Statistical significance for the differential HDX data is determined by an unpaired *t*-test for each time point, a procedure that is integrated into the HDX Workbench software. Corrections for back-exchange were made on the basis of an estimated 70% deuterium recovery and accounting for the known 80% deuterium content of the deuterium exchange buffer.

### Macrophage phagocytosis assay

CellTrace™ Far Red dye (Invitrogen)-labeled hMDMs were mixed with CellTrace™ Violet dye (Invitrogen)-labeled DLD-1 cells or Raji-hPD-L1 cells at a ratio of 1:2 (hMDM per tumor cell). In some cases, 20 nM of human IgG4 isotype control or serial dilutions of anti-SIRPα antibodies were added and incubated with the cell mixture of hMDM/DLD-1 for 2 h at 37°C. In some cases, 1 nM ESD05_2719, 10 nM human IgG4 isotype control, 10 nM anti-SIRPα antibodies with or without 1 nM human IgG1 isotype control, serial dilutions of anti-SIRPα antibodies with or without 1 nM ESD05_2719 were added and incubated with the cell mixture of hMDM/Raji-hPD-L1 for 2 h at 37°C. The cell mixtures were then analyzed by a flow cytometer (FACSCanto II, BD Biosciences). The percentage of CellTrace™ Far Red and CellTrace™ Violet double-positive macrophage population in total macrophages (CellTrace™ Far Red positive cells) was calculated as phagocytosis index to quantitate the activity of each sample to potentiate macrophage phagocytosis.

### Anti-CD3/CD28-induced T-cell activation assay

With or without the presence of 100 nM of test antibodies, CellTrace™ Violet dye (Invitrogen)-labeled T cells were treated with ImmunoCult™ Human CD3/CD28 T Cell Activator (Stemcell) for 4 days. IFN-γ levels in harvested culture supernatants were measured using a homogeneous time-resolved fluorescence (HTRF)–based human IFN-γ kit (Cisbio). Meanwhile, the proliferation of T cells were quantitated by detecting CellTrace™ Violet dye dilution. Briefly, cells were stained with Far Red fluorescent reactive dye (Invitrogen) for 15 min at RT to label dead cells. After washing twice with DPBS buffer, BV605 labeled anti-CD8 antibody (Biolegend), and APC/Cy7 labeled anti-CD4 antibody (Biolegend) were added and incubated with the cells for 30 min at 4°C. After washing twice with FACS buffer, cells were then analyzed by a flow cytometer (FACSCanto II, BD Biosciences). The Far Red fluorescent reactive dye unlabeled cells were live cells. The percentage of live CD4^+^ or CD8^+^ T cells with reduced levels of CellTrace™ Violet dye labeling in total live CD4^+^ or CD8^+^ T cells was calculated to quantitate the effect of each sample on T-cell proliferation. One-way ANOVA tests were performed using GraphPad Prism 9.0 to compare the data across different treatment groups.

### Allogeneic mixed lymphocyte reaction

In the presence of 5 μg/ml of Keytruda or 50 nM of test antibodies, allogeneic human MoDCs were cocultured with CellTrace™ Violet dye (Invitrogen)-labeled T cells at a ratio of 1:4 (MoDC per T cell). After 5 days of coculture, IFN-γ levels in harvested culture supernatants were measured using an HTRF-based human IFN-γ kit (Cisbio). Meanwhile, the proliferation of T cells were quantitated by the same method used in anti-CD3/CD28-induced T-cell activation assay. One-way ANOVA tests were performed using GraphPad Prism 9.0 to compare the data across different treatment groups.

**Figure 1:**
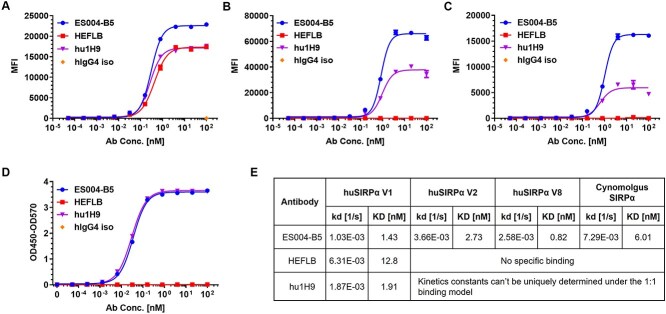
ES004-B5 is a high affinity pan-allelic and cynomolgus-cross-reactive anti-human SIRPα mAb. (A–C) Binding curves of ES004-B5 and reference antibodies to huSIRPα V1-expressing CHOK1/hSIRPα V1 cells (A), huSIRPα V2-expressing CHOK1/hSIRPα V2 cells (B), and cynomolgus SIRPα-expressing CHOK1/cSIRPα cells (C) as determined by FACS mean fluorescence intensity (MFI). (D) Binding curves of ES004-B5 and reference antibodies to recombinant huSIRPα V8 ECD protein as determined by ELISA. (E) Binding affinities and kinetics of ES004-B5 and reference antibodies to huSIRPα V1, V2, V8, and cynomolgus SIRPα as determined using SPR technique on a Biacore T200 instrument at 25°C. hIgG4 iso is a human IgG4 isotype control. In Fig. 1A–D, mean (*n* = 2) ± SD is shown.

### Syngeneic tumor models

The effect of ES004-B5 with or without ES0060028, an anti-huCLDN18.2 antibody (produced and described in this study), on inhibiting Claudin 18.2 expressing-tumor growth *in vivo* was assessed in a MC38-huCLDN18.2/huCD47 syngeneic tumor model at Shanghai Model Organisms (huSIRPα/huCD47 double knock-in C57BL/6 mice and the MC38-huCLDN18.2/huCD47 tumor cell line were generated by Shanghai Model Organisms). huSIRPα/huCD47 double knock-in C57BL/6 mice were implanted with 3 million MC38-huCLDN18.2/huCD47 cells subcutaneously in their right flank. Groups of eight mice were then dosed intraperitoneally with vehicle alone (0.9% NaCl), 10 mg/kg ES004-B5, 10 mg/kg ES0060028, or 10 mg/kg ES004-B5 plus 10 mg/kg ES0060028 twice per week for a total of four doses, starting once average tumor volume (TV) reached 80–90 mm^3^.

The effect of ES004-B5 was likewise tested with or without an anti-PD-L1 single-domain antibody, KN035 (see Antibodies and Reagents) using a CT26-huPD-L1/huCD47 syngeneic tumor model at GemPharmatech (huSIRPα/huPD-L1 double knock-in BALB/c mice and the CT26-huPD-L1/huCD47 tumor cell line were generated by GemPharmatech). huSIRPα/huPD-L1 double knock-in BALB/c mice were implanted with 2 million CT26-huPD-L1/huCD47 cells subcutaneously in their right flank. Groups of six mice were then dosed intraperitoneally with vehicle alone (0.9% NaCl), 10 mg/kg ES004-B5, 10 mg/kg KN035, or 10 mg/kg ES004-B5 plus 10 mg/kg KN035 twice per week for a total of six doses, starting when average TV reached 100 mm^3^.

In both studies, tumor sizes were measured twice per week and TVs were calculated with the formula: TV = 1/2 × length × width^2^. Relative TV (RTV) was obtained by calculating the ratio of TV post-treatment and pretreatment. Relative tumor growth inhibition rate (TGI%) was calculated with the formular: TGI% = (1 − T_RTV_/C_RTV_) × 100%. T_RTV_ and C_RTV_ are the mean relative TVs of the treatment groups and the vehicle group. Two-way ANOVA tests were performed using GraphPad Prism 9.0 to compare the mean TVs of the different treatment groups.

### *In vivo* MoA (mechanism of action) studies

In CD8 T-cell depletion *in vivo* study, huSIRPα/huCD47 double knock-in C57BL/6 mice (Biocytogen) were implanted with 3 million MC38-huCLDN18.2/huCD47 cells (Shanghai Model Organisms) subcutaneously in their right flank, and grouped when average TV reached 70–80 mm^3^. Tumor cell inoculated mice were intraperitoneally injected with 200 μg of mouse IgG2a isotype control (Leinco) or Ly2.2 (an anti-CD8a antibody, Leinco) on the day before grouping (Day −1), and on Days 4, 7, and 11 after grouping. One group of six mice injected with mouse IgG2a isotype control were dosed intraperitoneally with vehicle alone (0.9% NaCl) twice per week after grouping for a total of four doses. Groups of six mice injected with anti-CD8 antibody were dosed intraperitoneally with vehicle alone or 15 mg/kg ES004-B5 plus 15 mg/kg ES0060028 twice per week for a total of four doses.

In IFNγ blockade *in vivo* study, huSIRPα/huCD47 double knock-in C57BL/6 mice (Shanghai Model Organisms) were implanted with 3 million MC38-huCLDN18.2/huCD47 cells (Shanghai Model Organisms) subcutaneously in their right flank and grouped when average TV reached 60–70 mm^3^. Tumor cell–inoculated mice were intraperitoneally injected with 200 μg of mouse IgG1 isotype control (Selleckchem) or XMG1.2 (an anti-IFNγ antibody, Selleckchem) on the day before grouping (Day −1), and on Days 2, 5, and 8 after grouping. Groups of four to five mice injected with mouse IgG1 isotype control were dosed intraperitoneally with vehicle alone (0.9% NaCl), or 15 mg/kg ES004-B5 plus 15 mg/kg ES0060028 twice per week for a total of four doses. A group of four mice injected with anti-IFNγ antibody were dosed intraperitoneally with 15 mg/kg ES004-B5 plus 15 mg/kg ES0060028 twice per week for a total of four doses.

In both studies, tumor sizes were measured twice per week and TVs were calculated with the formula: TV = 1/2 × length × width^2^.

### Toxicity study in nonhuman primates

Placebo or ES004-B5 (15, 45, or 90 mg/kg) was administered to cynomolgus monkeys (five males and five females per group) by intravenous infusion once-weekly over 4 weeks (five doses in total). After the final dosing, two male and two female monkeys in each group were maintained for a 4-week recovery period. Data on mortality, clinical signs, body temperature, body weight, and food consumption were collected throughout the study. Blood samples for toxicokinetic analysis were collected at predose (Day 0), and 1 min, 1, 4, 24, 72, 120, and 168 h following the end of the infusion on Day 1 and Day 22. Blood samples for receptor occupancy analysis (on peripheral CD14^+^CD11b^+^ monocytes) were collected at predose (Day 0), Day 2, Day 8, Day 22, Day 23, Day 29, Day 36, Day 43, Day 50, and Day 57. Blood samples for hematology analysis and clinical chemistry analysis were collected at predose (10 and 3 days before dosing), Day 15, Day 29, and Day 57. At the end of the study, all monkeys were sacrificed for pathology examination (organ weights, macroscopic and microscopic examinations).

**Figure 2:**
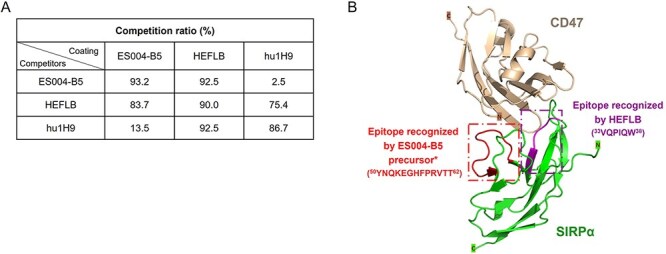
ES004-B5 binds to a unique epitope on the CD47-binding domain of SIRPα. (A) Competition between ES004-B5 and reference antibodies in binding to huSIRPα V1 ECD recombinant protein measured by an ELISA-based competition assay. (B) Structure of CD47 (wheat) complexed with SIRPα D1 domain (green). The epitopes of ES004-B5 precursor (red) and HEFLB (purple) on SIRPα D1 domain identified by HDX/MS were highlighted with different colors. ES004-B5 was humanized from ES004-B5 precursor, which is a chimeric human IgG4.

## Results

### ES004-B5 is a pan-allelic and cynomolgus-cross-reactive anti-human SIRPα mAb

ES004-B5, produced as described in Materials and Methods, strongly bound to huSIRPα variants V1 and V2 overexpressed on CHO-K1 cells (Fig. 1A and B) as well as recombinant huSIRPα variant V8 ECD protein (Fig. 1D) in a dose-dependent manner. Binding kinetic analysis showed that ES004-B5 bound to recombinant huSIRPα V1, V2, and V8 ECD proteins with KD values of 1.43 nM (huSIRPα V1), 2.73 nM (huSIRPα V2), and 0.82 nM (huSIRPα V8), respectively (Fig. 1E, Supplementary Fig. 1A). These data suggest that ES004-B5 is a high-affinity anti-SIRPα mAb for all major allelic variants of huSIRPα. ES004-B5 also bound to cynomolgus monkey SIRPα with high affinity, enabling the use of this NHP species for preclinical toxicity studies (Fig. 1C and E). In contrast, the reference antibody HEFLB bound only to huSIRPα V1 with lower affinity than that of ES004-B5, and it did not bind to huSIRPα V2, V8 and cynomolgus monkey SIRPα (Fig. 1, Supplementary Fig. 1B); another reference antibody hu1H9 is a high-affinity huSIRPα V1 antibody, but with a much weaker binding affinity to huSIRPα V2, V8, and cynomolgus SIRPα (Fig. 1, Supplementary Fig. 1C).

### ES004-B5 binds to a unique epitope on the CD47-binding domain of SIRPα

ES004-B5 did not compete with hu1H9 for binding to recombinant huSIRPα V1 ECD protein in ELISA, indicating that ES004-B5 has a unique antigen binding epitope distinct from that of hu1H9 (Fig. 2A). The binding epitopes of ES004-B5 precursor (ES004-B5 is a humanized antibody derived from ES004-B5 precursor) and HEFLB were mapped using HDX-MS. Deuteration in the peptide “YNQKEGHFPRVTTVSDL” was significantly reduced upon ES004-B5 precursor binding, indicating that this region in the SIRPα IgV domain is essential for ES004-B5 binding (Fig. 2B, Supplementary Fig. 2A). In contrast, HEFLB binding resulted in significantly less deuteration in a different peptide, “VGPIQW”, indicating that the binding epitope of HEFLB on SIRPα is different from that of ES004-B5 (Fig. 2B, Supplementary Fig. 2B). However, the spatial location of these two epitopes is apparently close enough to result in steric hindrance between antibodies because ES004-B5 competed with HEFLB for binding to recombinant huSIRPα V1 ECD protein (Fig. 2A).

**Figure 3:**
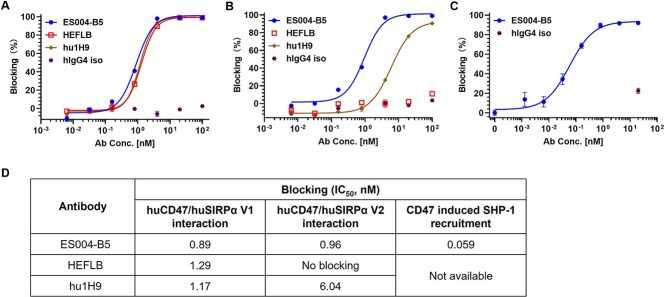
ES004-B5 is a potent SIRPα blocker. (A, B) Titration curves of ES004-B5 and reference antibodies blocking huCD47 ECD recombinant protein binding to huSIRPα V1-expressing CHOK1/hSIRPα V1 cells (A) or huSIRPα V2-expressing CHOK1/hSIRPα V2 cells (B) as determined in FACS-based competition assays. (C) Titration curve of ES004-B5 blocking CD47-induced recruitment of SHP-1 to SIRPα intracellular tail as determined in SIRPα/SHP-1 recruitment assay. (D) IC_50_ of ES004-B5 and reference antibodies in competition assays and SIRPα/SHP-1 recruitment assay calculated by GraphPad Prism 9.0 using four-parameter nonlinear fitting. hIgG4 iso is a human IgG4 isotype control. In Fig. 3A and B, mean (*n* = 2) ± SD is shown. In Fig. 3C, mean (*n* = 3) ± SD is shown.

**Figure 4:**
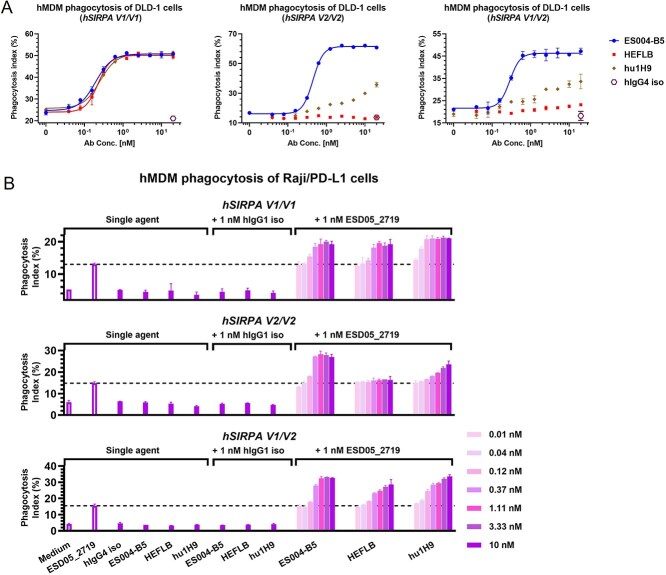
ES004-B5 promotes phagocytosis of tumor cells by macrophages of all *SIRPA* genotypes. (A, B) ES004-B5 promoted the phagocytosis of DLD-1 cells (A) and ESD05_2719 opsonized Raji-hPD-L1 cells (B) by hMDMs of *SIRPA V1/V1 SIRPA V2/V2* or *SIRPA V1/V2* genotype. ESD05_2719 is an anti-PD-L1 antibody. hIgG4 iso is a human IgG4 isotype control. hIgG1 iso is a human IgG1 isotype control. *SIRPA* genotypes of hMDM donors are listed at the top of each panel. Mean (*n* = 2) ± SD is shown.

### ES004-B5 potently blocks CD47/SIRPα interaction and CD47-induced SIRPα signaling

The epitope mapping results revealed that ES004-B5 recognizes the C’D loop in the SIRPα IgV domain, a region known to contribute to SIRPα interaction with CD47. Thus, the ability of ES004-B5 to block CD47/SIRPα interaction was then assessed in FACS-based competition assays. ES004-B5 completely blocked recombinant huCD47 ECD binding to huSIRPα V1-expressing cells and huSIRPα V2-expressing cells (Fig. 3A, B, and D).

Upon ligation with SIRPα, CD47-induced “don't-eat-me” signaling is transmitted via phosphorylation of the ITIMs in the SIRPα cytoplasmic tail. Subsequent binding and activation of both SHP-1 and SHP-2 block phagocytosis, potentially by preventing accumulation of myosin-IIA at the phagocytic synapse. Based on this signal transduction mechanism, a reporter assay was developed, based on SIRPα/SHP-1 recruitment, to assess ability of anti-SIRPα mAbs to prevent CD47-induced SIRPα signaling (Supplementary Fig. 3). ES004-B5 effectively disrupted CD47-induced recruitment of SHP-1 to the SIRPα cytoplasmic tail with an IC_50_ of 0.059 nM (Fig. 3C and D). These data suggest that ES004-B5 is a potent CD47/SIRPα interaction blocker that can effectively neutralize CD47-induced SIRPα signaling. In contrast, although HEFLB and hu1H9 were comparable to ES004-B5 in blocking CD47/SIRPα V1 interaction (Fig. 3A and D), they showed much weaker or no blocking activity on huSIRPα V2 (Fig. 3B and D).

### ES004-B5 effectively promotes phagocytosis of tumor cells by macrophages of all *SIRPA* genotypes

ES004-B5 effectively promoted phagocytosis of DLD-1 colon tumor cells and ESD05_2719 (an anti-PD-L1 antibody)-opsonized Raji-hPD-L1 lymphoma cells by hMDMs from *SIRPA V1/V1* homozygous*, V2/V2* homozygous, and *V1/V2* heterozygous genotypes (Fig. 4A and B, Table 1). Likewise, ES004-B5 also effectively promoted hMDM-mediated phagocytosis of rituximab-opsonized Raji lymphoma cells and cetuximab-opsonized HCT116 colon tumor cells (Supplementary Fig. 4A and B). These data suggest that ES004-B5 can potently potentiate human macrophages of all *SIRPA* genotypes to phagocytose tumor cells and tumor cells opsonized by antibodies targeting TAAs via blocking CD47/SIRPα “don't-eat-me” signaling. In contrast, although HEFLB and hu1H9 were comparable to ES004-B5 in potentiating human macrophages of *SIRPA V1/V1* genotype to phagocytose tumor cells, they have weaker or no binding and blocking activity to huSIPRα V2 and therefore showed no or weaker effect on human macrophages of *SIRPA V2/V2* and *SIRPA V1/V2* genotypes (Fig. 4A and B, Table 1, Supplementary Fig. 4A)*.*

### ES004-B5 binds SIRPγ but does not negatively impact T-cell activation

**Figure 5:**
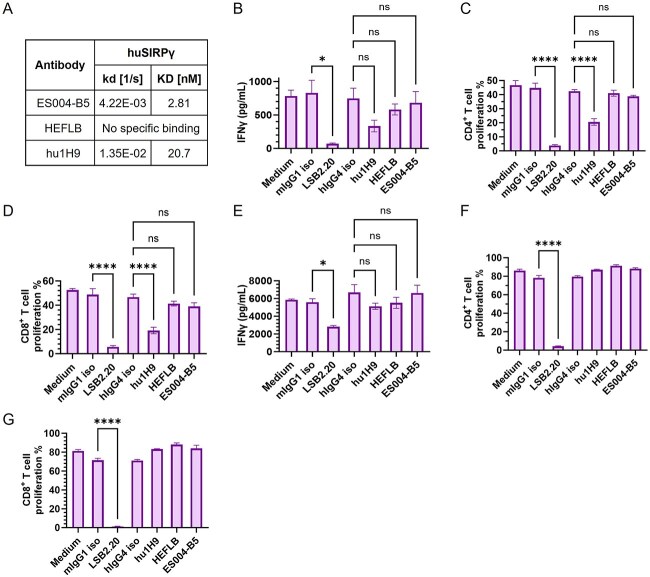
ES004-B5 does not negatively impact T-cell activation. (A) Binding affinities and kinetics of ES004-B5 and reference antibodies to huSIRPγ as determined using SPR technique on a Biacore T200 instrument at 25°C. (B-G) ES004-B5 did not significantly alter IFN-γ release by T cells (B) as judged after 4 days of anti-CD3/CD28 antibody-mediated stimulation, nor did it significantly reduce CD4^+^ T cell (C) or CD8^+^ T cell (D) proliferation as judged by CellTrace™ Violet dye dilution. Likewise, ES004-B5 also did not significantly alter allogeneic MoDC-stimulated CD4^+^ T-cell proliferation (F), CD8^+^ T-cell proliferation (G) and IFN-γ release by T cells (E). hIgG4 iso is a human IgG4 isotype control. mIgG1 iso is a mouse IgG1 isotype control. LSB2.20 is a SIRPγ-blocking antibody. Mean (*n* = 3) ± SD is shown. One-way ANOVA tests were performed using GraphPad Prism 9.0 to compare the data across different treatment groups. ^*^P-value < 0.05, ^*^^*^^*^^*^P-value < 0.0001.

Signal regulatory protein gamma (SIRPγ; also known as SIRP beta2) is another important member of the SIRP family, which is highly expressed on T cells [37]. It was reported that adhesion of human T cells to antigen-presenting cells through SIRPγ-CD47 interaction costimulates T-cell proliferation [38]. Although ES004-B5 has high binding affinity to huSIRPγ (Fig. 5A, Supplementary Fig. 5), it did not significantly alter anti-CD3/CD28 antibodies- or allogeneic MoDC-stimulated T-cell proliferation (Fig. 5C, D, F, and G), nor did it significantly reduce IFN-γ release by T cells (Fig. 5B and E). As a positive control, the SIRPγ blocking antibody LSB2.20 significantly suppressed T-cell proliferation and IFN-γ release in T-cell activation assays (Fig. 5B–G). The reference antibody hu1H9 binds to huSIRPγ with weaker affinity than ES004-B5, and HEFLB does not recognize huSIRPγ (Fig. 5A, Supplementary Fig. 5). As expected, HEFLB did not show any negative impact in T-cell activation assays (Fig. 5B–G). However, hu1H9 suppressed allogeneic MoDC-stimulated T-cell activation (Fig. 5B–D). Collectively, these data suggest that ES004-B5 does not negatively impact T-cell activation.

**Table 1:** hMD phagocytosis of DLD-1 cells or anti-PD-L1-opsonized Raji-hPD-L1 cells in the presence of anti-SIRPα antibodies.

**Target cells: DLD-1**									
**Antibody**M	**Donor 1 (*SIRPA V1/V1*)**			**Donor 2 (*SIRPA V2/V2*)**			**Donor 3 (*SIRPA V1/V2*)**		
	**EC**_**50,**_ **[nM]**	**TOP^b^** **(%)**	**Window^c^** **(%)**	**EC**_**50,**_ **[nM]**	**TOP^b^** **(%)**	**Window^c^** **(%)**	**EC**_**50,**_ **[nM]**	**TOP^b^** **(%)**	**Window^c^** **(%)**
ES004-B5	0.19	50.8	27.0	0.42	61.9	45.2	0.29	46.3	25.3
HEFLB	0.22	50.0	26.3	No activity					
hu1H9	0.17	51.1	27.3	Weak activity					
**Target cells: ESD05_2719 (anti-PD-L1) opsonized Raji-hPD-L1**									
**Antibody**	**Donor 1 (*SIRPA V1/V1*)**			**Donor 2 (*SIRPA V2/V2*)**			**Donor 3 (*SIRPA V1/V2*)**		
	**EC**_**50,**_ **[nM]**	**TOP^b^** **(%)**	**Window** ^a^ **(%)**	**EC**_**50,**_ **[nM]**	**TOP^b^** **(%)**	**Window** ^a^ **(%)**	**EC**_**50,**_ **[nM]**	**TOP^b^** **(%)**	**Window** ^a^ **(%)**
ES004-B5	0.16	19.5	6.5	0.16	27.8	13.0	0.25	32.9	17.4
HEFLB	0.19	19.2	6.1	No activity			0.30	28.4	12.9
hu1H9	0.02	21,2	8.2	Weak activity			0.14	33.4	17.9

EC_50_ and TOP phagocytosis index of ES004-B5 and reference antibodies in macrophage phagocytosis assays were calculated by GraphPad Prism 9.0 using four-parameter nonlinear fitting.

^a^Phagocytosis window = TOP phagocytosis index − Phagocytosis index _1 nM ESD05_2719_

^b^TOP phagocytosis index

^c^Phagocytosis window = TOP phagocytosis index − Phagocytosis index _medium_

### ES004-B5 enhances antitumor activity in combination with anti-tumor-associated antigen antibodies in syngeneic tumor models

As ES004-B5 does not bind to murine SIRPα, human SIRPα knock-in mice were used to study the antitumor effect of ES004-B5 with or without anti-TAA antibodies in syngeneic tumor models.

**Figure 6:**
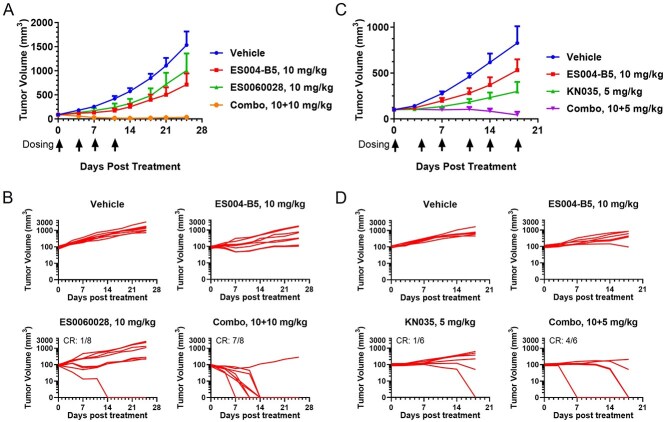
ES004-B5 enhances antitumor activity in combination with anti-TAA antibodies in syngeneic tumor models. (A, B) huSIRPα/huCD47 double knock-in C57BL/6 mice were implanted with MC38-huCLDN18.2/huCD47 cells and treated with vehicle alone (0.9% NaCl), 10 mg/kg ES004-B5, 10 mg/kg ES0060028, or 10 mg/kg ES004-B5 plus 10 mg/kg ES0060028 twice per week for a total of 4 doses (*n* = 8), starting when average tumor sizes were 80–90 mm^3^. Mean TV growth curves for each group are shown in (A) and tumor growth curves for individual mice are shown in (B). (C, D) huSIRPα/huPD-L1 double knock-in BALB/c mice were implanted with CT26-huPD-L1/huCD47 cells and treated with vehicle alone (0.9% NaCl), 10 mg/kg ES004-B5, 5 mg/kg KN035, or 10 mg/kg ES004-B5 plus 5 mg/kg KN035 twice per week for a total of six doses (*n* = 6), starting when average TV reached 100 mm^3^. Mean TV growth curves for each group are shown in (C), and tumor growth curves for individual mice are shown in (D). In Fig. 6A and C, mean ± SEM is shown.

In the efficacy study to investigate the effect of ES004-B5 in combination with ES0060028, an anti-huCLDN18.2 antibody, huSIRPα/huCD47 double knock-in C57BL/6 mice (Shanghai Model Organisms) were implanted with MC38-huCLDN18.2/huCD47 tumor cells (Shanghai Model Organisms; mouse CD47 replaced with human CD47, huCLDN18.2-expressing) and randomized into four treatment groups with an average TV of 80–90 mm^3^ before initiation of treatment. At the end of the study (25 days after treatment), ES004-B5 monotherapy, anti-huCLDN18.2 antibody monotherapy, and the combination of ES004-B5 with anti-huCLDN18.2 antibody reduced tumor growth by 55.07%, 35.41%, and 97.57%, respectively (Fig. 6A). Tumors disappeared in one of eight mice after anti-huCLDN18.2 antibody monotherapy and in seven of eight mice after the combination therapy (Fig. 6B). The combination therapy was superior to ES004-B5 or anti-huCLDN18.2 antibody monotherapy in suppressing MC38-huCLDN18.2/huCD47 tumor growth (Supplementary Table 1). Three months after the end of the efficacy study, six mice cured after the combination therapy were rechallenged with MC38-huCLDN18.2/huCD47 cells. None of them developed tumors, suggesting that the combination therapy induced immune memory.

In the efficacy study to investigate the effect of ES004-B5 in combination with KN035, an anti-PD-L1 antibody, huSIRPα/huPD-L1 double knock-in BALB/c mice (GemPharmatech) were implanted with CT26-huPD-L1/huCD47 tumor cells (GemPharmatech; mouse CD47 replaced with human CD47, huPD-L1-expressing) and randomized into four treatment groups with an average TV of 100 mm^3^ before initiation of treatment. At the end of the study (18 days after treatment), ES004-B5 monotherapy, KN035 monotherapy, and the combination of ES004-B5 with KN035 reduced tumor growth by 38.13%, 65.71%, and 94.69%, respectively (Fig. 6C). Tumors disappeared in one of six mice after KN035 monotherapy and in four of six mice after the combination therapy (Fig. 6D). The combination therapy was superior to ES004-B5 or KN035 monotherapy in suppressing CT26-huPD-L1/huCD47 tumor growth (Supplementary Table 2).

### T cells contribute to ES004-B5/anti-tumor-associated antigen combination therapy–induced inhibition of tumor growth

**Figure 7:**
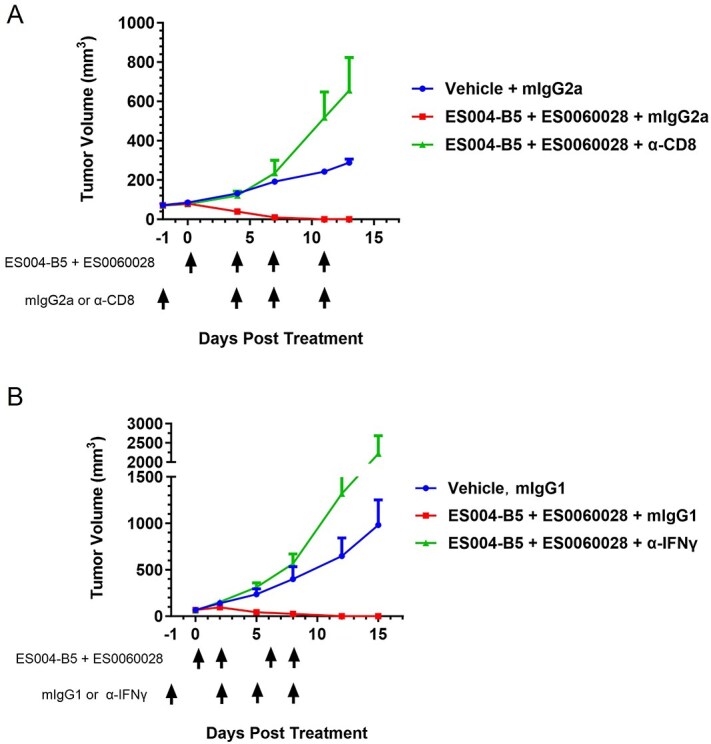
T cells contribute to ES004-B5/anti-TAA combination therapy–induced inhibition of tumor growth. (A) huSIRPα/huCD47 double knock-in C57BL/6 mice were implanted with MC38-huCLDN18.2/huCD47 cells and treated with vehicle (0.9% NaCl) plus control IgG, ES004-B5/ES0060028 plus control IgG, or ES004-B5/ES0060028 plus an anti-CD8 antibody according to the indicated schedule (bottom panel). TVs were measured at the indicated time points, and mean TV growth curves for each group are shown in top panel. mIgG2a is a mouse IgG2a isotype control. (B) huSIRPα/huCD47 double knock-in C57BL/6 mice were implanted with MC38-huCLDN18.2/huCD47 cells and treated with vehicle (0.9% NaCl) plus control IgG, ES004-B5/ES0060028 plus control IgG, or ES004-B5/ES0060028 plus an anti-IFNγ antibody according to the indicated schedule (bottom panel). TVs were measured at the indicated time points and mean TV growth curves for each group are shown in top panel. mIgG1 is a mouse IgG1 isotype control.

To investigate whether immune cells other than macrophages participate in the inhibition of tumor growth by ES004-B5 in combination with anti-TAA antibodies *in vivo*, we first examined the effect of ES004-B5/ES0060028 combination therapy on the population of immune cells in tumors formed by implanted MC38-huCLDN18.2/huCD47 cells. TIL analysis after 5 days of treatment of ES004-B5 in combination with ES0060028 showed an increase in the frequency of CD8 T cells, while there is a decrease in the frequency of M2 macrophages (F4/80^+^CD206^high^), thereby confirming that the TME was modified after the combination therapy (Supplementary Fig. 6). We next examined whether CD8 T cells contribute to the inhibition of tumor growth by ES004-B5/ES0060028 combination therapy. Treatment of huSIRPα/huCD47 double knock-in C57BL/6 mice with an mAb against CD8α effectively depleted CD8 T cells from the spleen and significantly attenuated the antitumor effect of the combination therapy for MC38-huCLDN18.2/huCD47 cells (Fig. 7A). Furthermore, we found that blocking IFNγ with an anti-IFNγ mAb completely attenuated the antitumor effect of the combination therapy (Fig. 7B). These results suggested that CD8 T cells and IFNγ are essential for the antitumor effect of ES004-B5/anti-TAA combination therapy.

### ES004-B5 demonstrates favorable pharmacokinetic properties and safety profile in cynomolgus monkeys

**Figure 8:**
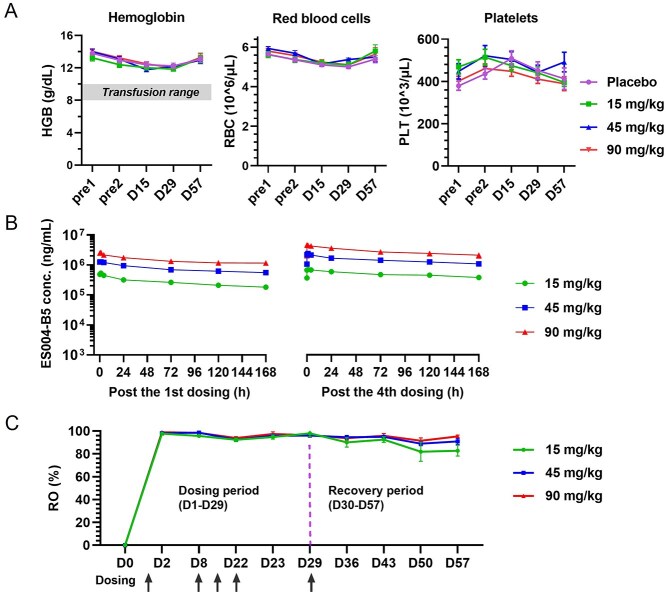
ES004-B5 demonstrates favorable pharmacokinetic properties and safety profile in cynomolgus monkeys. Placebo or ES004-B5 (15, 45, or 90 mg/kg) was administered to cynomolgus monkeys by intravenous infusion once-weekly over 4 weeks (*n* = 10, 5 males, 5 females). Hemoglobin/erythrocytes/platelets levels (A), serum concentration of ES004-B5 (B), and receptor occupancy on peripheral blood CD14^+^CD11b^+^ monocytes (C) were detected at the indicated time points. Mean ± SEM is shown. Pre1: 10 days before dosing; Pre2: 3 days before dosing. The shaded bar in (A) indicates the range of hemoglobin typically requiring a transfusion in human.

ES004-B5 binds to human SIRPα and cynomolgus SIRPα with comparable affinity (Fig. 1E). Thus, a GLP-compliant 4-week repeat-dose toxicity study was conducted in cynomolgus monkeys to investigate the pharmacokinetic and toxicological profile of ES004-B5. Administration of ES004-B5 by intravenous infusion was well tolerated in all monkeys at all dose levels tested (15, 45, and 90 mg/kg/dose). No abnormal clinical signs or ES004-B5-related changes in body temperature, body weight, or food consumption were observed. There were also no ES004-B5-related changes in clinical chemistry or hematology detected. No transient anemia or thrombocytopenia was detected with ES004-B5 infusion compared to the placebo control group (Fig. 8A). In addition, no ES004-B5-related adverse effects in gross pathology, relative organ weights, or histopathology were observed. These data indicate that ES004-B5 has a favorable safety profile as compared to CD47-targeted agents.

Serum exposure of ES004-B5 was measured and analyzed after the first and fourth dosing (Fig. 8B). The maximum concentration (*C*_max_) and area under the concentration–time curve (AUC) increased proportionally over the dose range of 15–90 mg/kg (Table 2). After 4 weeks of once-weekly intravenous administration, accumulation of ES004-B5 in serum was observed, with mean accumulation ratios ranging from 1.24 to 2.24 for *C*_max_ and from 1.41 to 2.85 for AUC_0–168h_ (Fig. 8B). At all dose levels, full receptor occupancy on peripheral blood CD14^+^CD11b^+^ monocytes was seen quickly after the first dose and remained unchanged throughout the whole dosing period (Fig. 8C). At the end of the recovery stage, receptor occupancy still remained above 80% at all dose levels (Fig. 8C).

## Discussion

Accumulating evidence suggests that the CD47-SIRPα “don't-eat-me” signaling axis is a prominent innate immune checkpoint. The development of anti-CD47 antibodies such as CC-90002, SRF-231, and magrolimab were discontinued likely due to severe side effects including grade 3 or 4 anemia, neutropenia, and thrombocytopenia [32–34]. SIRPα-Fc fusion protein TTI-621 also triggered grade 4 transient thrombocytopenia and had a relatively low maximum tolerated dose (MTD, 0.2 mg/kg) [31]. Although a priming/maintenance dosing strategy did reduce anemia incidence in the case of another anti-CD47 antibody, magrolimab [27, 29], the phase 3 ENHANCE trial in which magrolimab was combined with venetoclax and azacytidine (to treat acute myeloid leukemia and myelodysplastic syndrome) was halted due to unexpected serious adverse reactions, suggesting that the strategy of using a “priming” dose followed by maintenance dosing cannot fully resolve the safety issues. Universal expression of CD47 also leads to a severe “antigen sink” effect of CD47-targeted agents, which cannot be relieved by optimizing dosing strategy.

Unlike CD47, SIRPα is mainly expressed on myeloid cells. Similar to the anti-SIRPα antibody, ES004-B5 produced and characterized here, multiple anti-SIRPα antibodies were reported to have favorable safety profiles in NHPs [37, 40] and are predicted to not have hematological adverse effects in clinical trials. Indeed, the first-in-class anti-SIRPα antibody BI 765063 (OSE-172) was well tolerated in human trials with no dose-limiting toxicities, and clinical efficacy was observed with BI 765063 both as a monotherapy and in combination with ezabenlimab (a PD-1 inhibitor) in solid tumors [35, 36]. These preclinical and clinical findings strongly suggest that SIRPα-targeted therapy will overcome limitations of anti-CD47 antibodies or SIRPα fusion proteins encountered in clinical settings.

Analysis of the distribution and frequency of SIRPα polymorphisms indicates that V1, V2, and V8 are the main SIRPα variants in human [37, 41]. The human *SIRPA* reference allele *SIRPA V1* is dominant in Europeans (63%), Africans (69.5%), Admixed American (53.8%), and South Asians (53%), whereas *SIRPA V2* dominates in East Asians (41.3%) [37, 41]. In these human populations, the frequency of *SIRPA V8* is around 13.6%–20.5% [37, 41]. Human SIRPα variant V2 and V8 differ from huSIRPα V1 by 13 and 8 amino acids, respectively, in their IgV domain. ES004-B5 binds to an epitope on the CD47 binding domain of SIRPα, which is conserved among the major allelic variants of SIRPα. ES004-B5 binds to huSIRPα V1, V2, and V8 with high affinity and can potently block the interactions of huCD47 with huSIRPα V1 and V2. In *in vitro* phagocytosis assays, ES004-B5 effectively promoted phagocytosis of tumor cells and tumor cells opsonized by antibodies targeting TAAs by hMDMs from *SIRPA V1/V1* homozygous*, V2/V2* homozygous, and *V1/V2* heterozygous genotypes. Therefore, it is expected that ES004-B5 has the potential to be effective in a broad range of patients. In contrast, the huSIRPα V1-specific antibody, HEFLB, or the huSIRPα V2 low-binding antibody, hu1H9, may have no or weaker effect on patients with *SIRPA V2/V2* and *SIRPA V1/V2* genotypes.

**Table 2:** Dose-proportional increase in serum exposure of ES004-B5 after repeat dosing in cynomolgus monkeys.

**Study day**	**Sex**	**Dosage (mg/kg/dose)**	**Dose** **ratio**	***C***_**max**_ **ratio**	**AUC**_**0–168h**_ **ratio**	***C***_**max**_ **(μg/ml)**	**AUC**_**0–168 h**_ (h^*^μg/ml)
After the first dosing	Male	15	1	1.0	1.0	601 ± 161	46 300 ± 2710
		45	3	2.5	2.9	1490 ± 374	132 000 ± 15 100
		90	6	4.7	5.7	2830 ± 330	263 000 ± 31 400
	Female	15	1	1.0	1.0	525 ± 103	41 800 ± 4650
		45	3	2.4	2.8	1270 ± 162	118 000 ± 13 700
		90	6	4.7	5.1	2460 ± 424	213 000 ± 20 700
After the fourth dosing	Male	15	1	1.0	1.0	826 ± 96.9	94 500 ± 11 600
		45	3	2.9	2.6	2400 ± 502	241 000 ± 99 100
		90	6	5.2	3.9	4300 ± 894	372 000 ± 186 000
	Female	15	1	1.0	1.0	650 ± 79.5	71 600 ± 19 600
		45	3	3.7	3.4	2390 ± 487	243 000 ± 68 000
		90	6	8.5	8.5	5510 ± 755	607 000 ± 71 900

Macrophage phagocytosis is governed by both “eat-me” and “don't-eat-me” signals. In the absence of antibody opsonization, we found that blocking the CD47-SIRPα “don't-eat-me” signaling axis by ES004-B5 alone was sufficient to promote macrophage phagocytosis of DLD cells, suggesting that DLD cells have some intrinsic prophagocytic signal. With other target cells tested, ES004-B5 exerted enhanced antitumor activity when used in combination with anti-TAA antibodies including anti-CD20, anti-PD-L1, anti-EGFR, and anti-CLDN18.2, which can trigger a FcγR-mediated “eat-me” signaling specifically toward TAA-expressing tumor cells. We also found that both CD8 T cells and IFNγ were essential for the antitumor effect of ES004-B5/anti-CLDN18.2 combination therapy in mouse tumor models. Furthermore, mice cured by ES004-B5/anti-CLDN18.2 combination therapy did not develop cancer again after being rechallenged by the same tumor cells. These results suggest that ES004-B5/anti-TAA combination therapy enhances myeloid cell phagocytosis of TAA-expressing tumor cells and subsequently induced an effective antitumor T-cell response. Considering that PD-L1 inhibitors block PD-1/PD-L1 T-cell immune checkpoints, it is reasonable to speculate that ES004-B5/anti-PD-L1 and other ES004-B5/anti-TAA combination therapies have both overlapping and distinct mechanisms in inhibiting tumor growth. More studies are needed to elucidate how ES004-B5/anti-TAA combination therapies cross-prime antitumor T-cell responses.

Recently, it was reported that the nonblocking anti-SIRPα antibody AL008 can enhance hMDM phagocytosis of tumor cells in the absence of opsonizing agents by simultaneously triggering the degradation of SIRPα and stimulating FcγR activation [42]. In the hybridoma campaign of developing ES004-B5, we also found that some nonblocking anti-SIRPα antibodies exerted function by triggering the degradation of SIRPα or some unknown noncompetitive mechanism (data not shown). Although these nonblocking anti-SIRPα antibodies demonstrated more potent single-agent effect in increasing tumor cell engulfment by macrophages, it is not clear yet whether the ability to directly stimulate macrophage activation in the absence of opsonizing agents might bring any safety concern in clinical use.

The mechanism of how SIRPγ-CD47 interaction regulates T-cell function remains poorly understood. Clinical-stage anti-SIRPα antibodies, such as CC-95251 (Bristol Myers Squibb), FSI-189 (Gilead), ADU-1805 (Exelixis), and BR105 (Bioray), all bind to SIRPγ to varying degrees. Since ES004-B5 has high binding affinity to huSIRPγ as well as SIRPα, we assessed whether it alters T-cell function using allogeneic MLR and anti-CD3/CD28-induced T-cell activation assays. ES004-B5 did not significantly alter magnitude of anti-CD3/CD28 antibody- or allogeneic MoDC-stimulated T-cell proliferation, nor did it alter IFN-γ release by T cells. As reported by others [38], the SIRPγ-blocking antibody LSB2.20 significantly suppressed T-cell activation in both assays. Interestingly, we found that hu1H9 binds to huSIRPγ with weaker affinity than ES004-B5 does, but unlike ES004-B5, hu1H9 did suppress allogeneic MoDC-stimulated T-cell activation. ES004-B5 has a unique antigen-binding epitope on SIRPα that is distinct from that of hu1H9. Presumably, they also bind to different regions on SIRPγ, resulting in functional differences in altering SIRPγ-mediated signaling and T-cell activation.

In summary, ES005-B5 is a potentially best-in-class SIRPα-targeted therapeutic antibody with a unique epitope that confers superior pan-allelic SIRPα binding and blocking activity. The combination of ES004-B5 with opsonizing agents exerts superior antitumor activity *in vitro* and *in vivo*, suggesting that ES004-B5 has potential to be an important backbone of SIRPα-based combination therapies or bispecific antibodies. Based on preclinical data herein, ES004-B5 may also have a favorable safety profile in human. Thus, these data support further advancement of ES004-B5 into clinical development.

## Supplementary Material

Supplementary_material_tbae022

## Data Availability

The data from this study are available from the corresponding author upon reasonable request.

## Animal research

The mouse *in vivo* efficacy studies were conducted at Shanghai Model Organisms Center and GemPharmatech. The mouse *in vivo* MoA studies were conducted at Shanghai Model Organisms Center Facility. The study protocols were reviewed and approved by CROs’ Institutional Animal Care and Use Committee (IACUC). The care and use of animals for experiments were performed in accordance with the Association for Assessment and Accreditation of Laboratory Animal Care (AAALAC). The NHPs toxicity study was conducted at Wuxi Apptec (Suzhou, China) in accordance with the US Food and Drug Administration Good Laboratory Practice (GLP) Regulations, 21 Code of Federal Regulations Part 58. The study plan was reviewed and approved by the Institutional Animal Care and Use Committee (IACUC) in compliance with the Association for Assessment and Accreditation of Laboratory Animal Care (AAALAC) International guidelines as reported in the Guide for the Care and Use of Laboratory Animals (National Research Council, 2011) and Regulations for the Administration of Affairs Concerning Experimental Animals (The People’s Republic of China, Ministry of Science & Technology, 2017).
