# Supplementary figures and images for: A pan-allelic human SIRPα-blocking antibody, ES004-B5, promotes tumor killing by enhancing macrophage phagocytosis and subsequently inducing an effective T-cell response
Source: Antib Ther. 2024 Aug 28;7(3):266–80. doi: 10.1093/abt/tbae022 (PMC11384143; doi:10.1093/abt/tbae022)

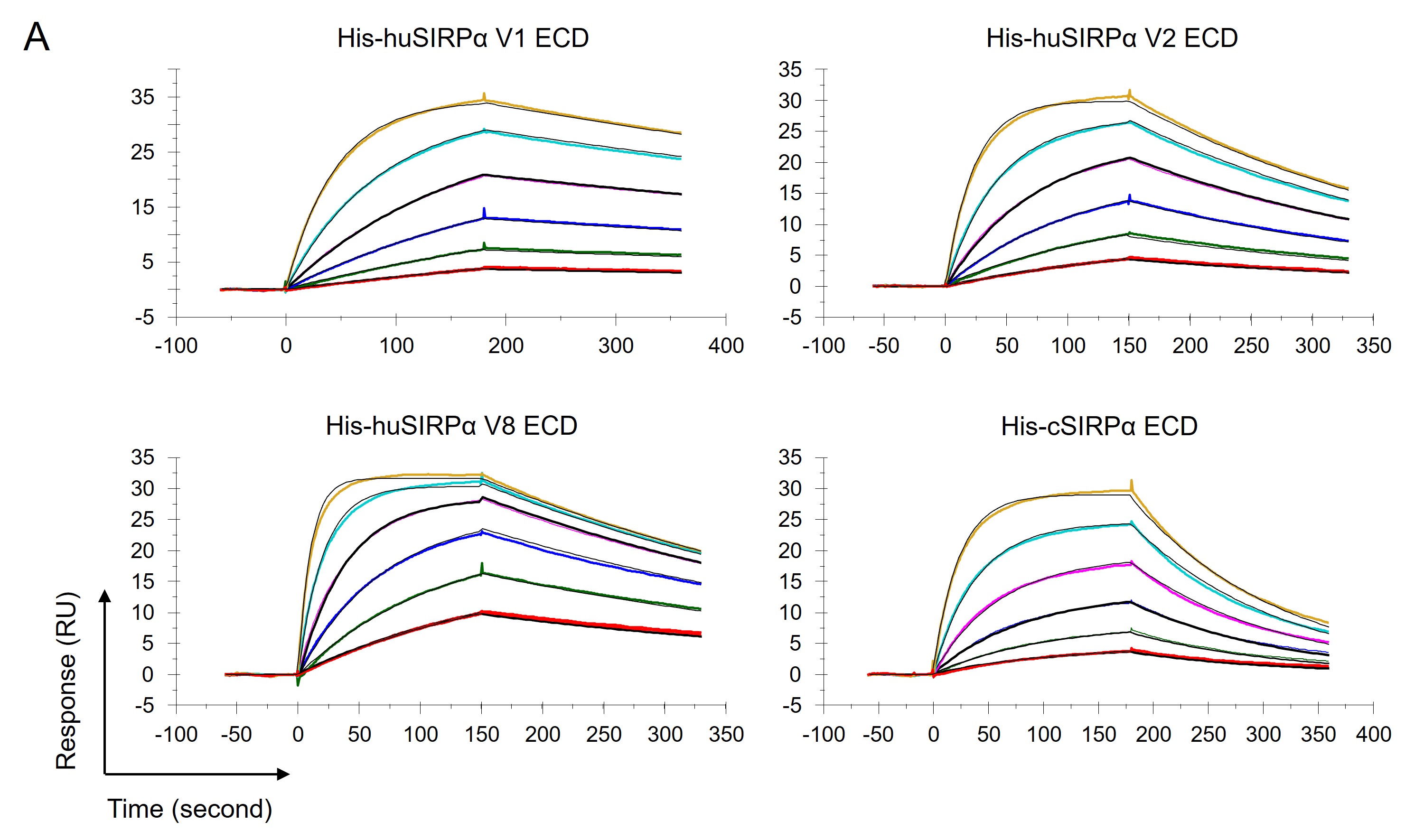

Supplement: Supplementary_material_tbae022 [file supplementary_material_tbae022.zip › Supplemental Figure 1A.jpg]

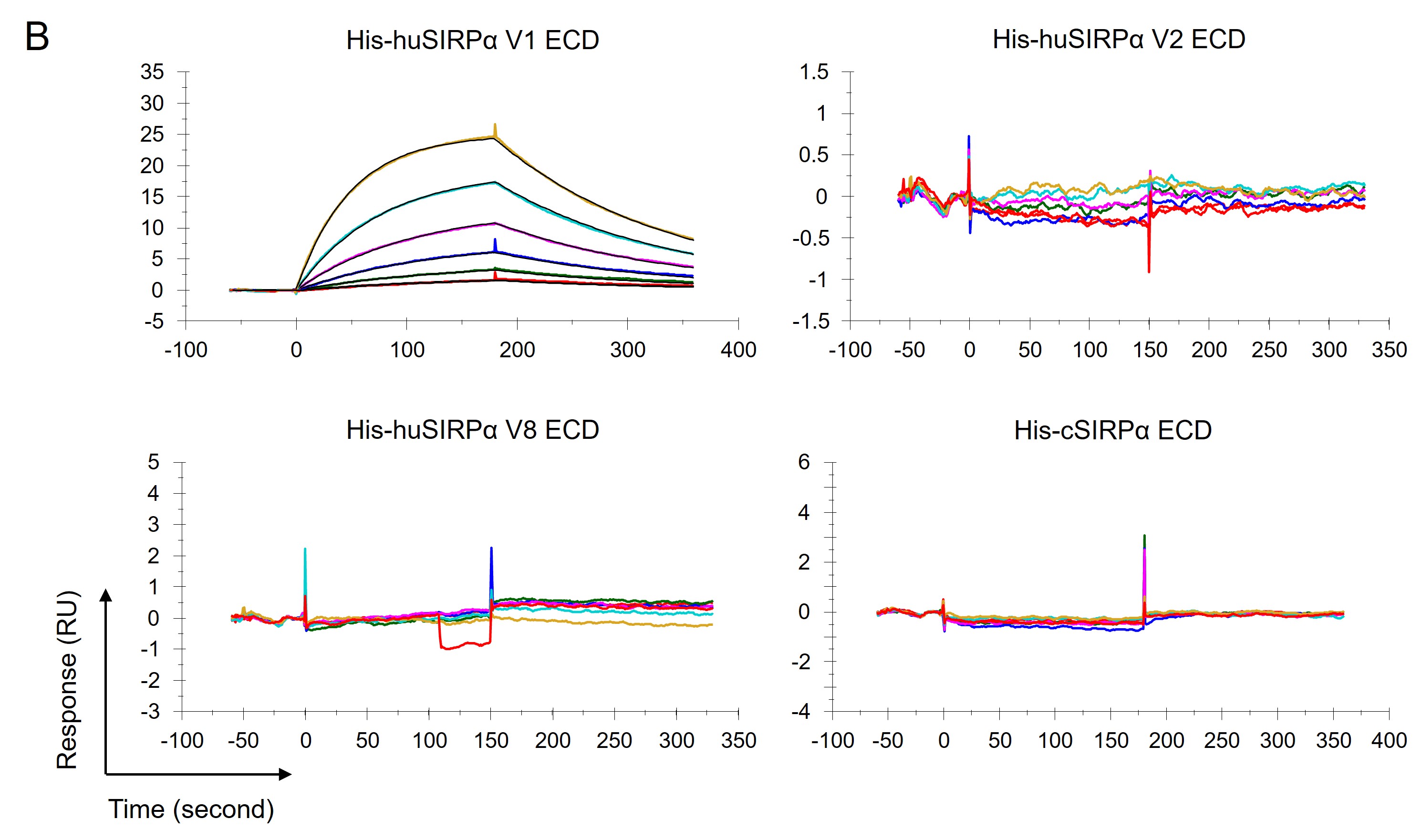

Supplement: Supplementary_material_tbae022 [file supplementary_material_tbae022.zip › Supplemental Figure 1B.jpg]

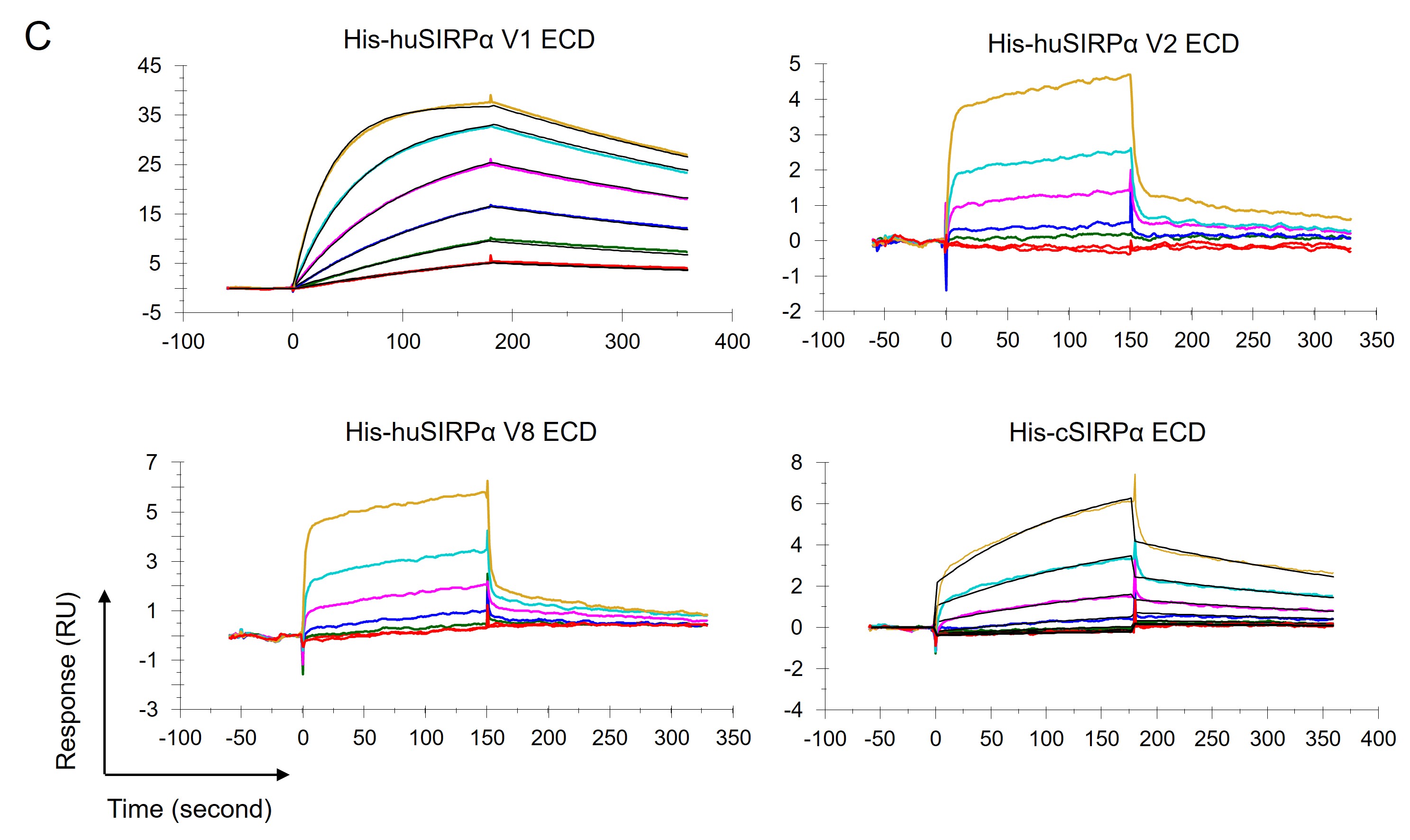

Supplement: Supplementary_material_tbae022 [file supplementary_material_tbae022.zip › Supplemental Figure 1C.jpg]

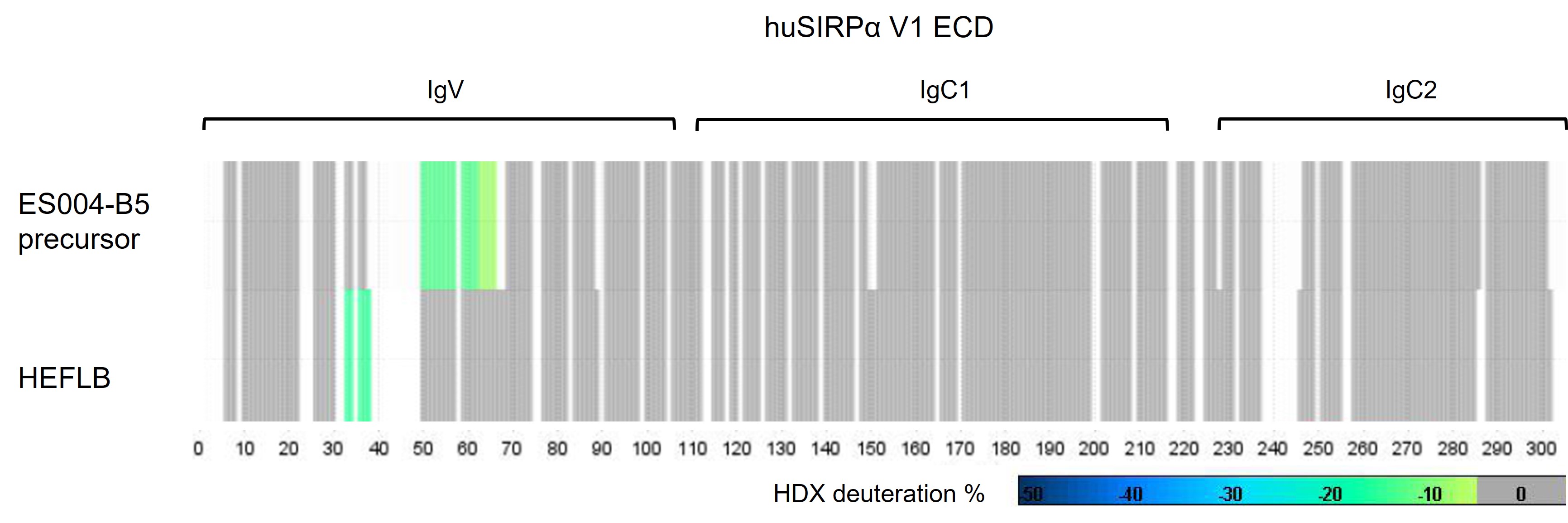

Supplement: Supplementary_material_tbae022 [file supplementary_material_tbae022.zip › Supplemental Figure 2A.jpg]

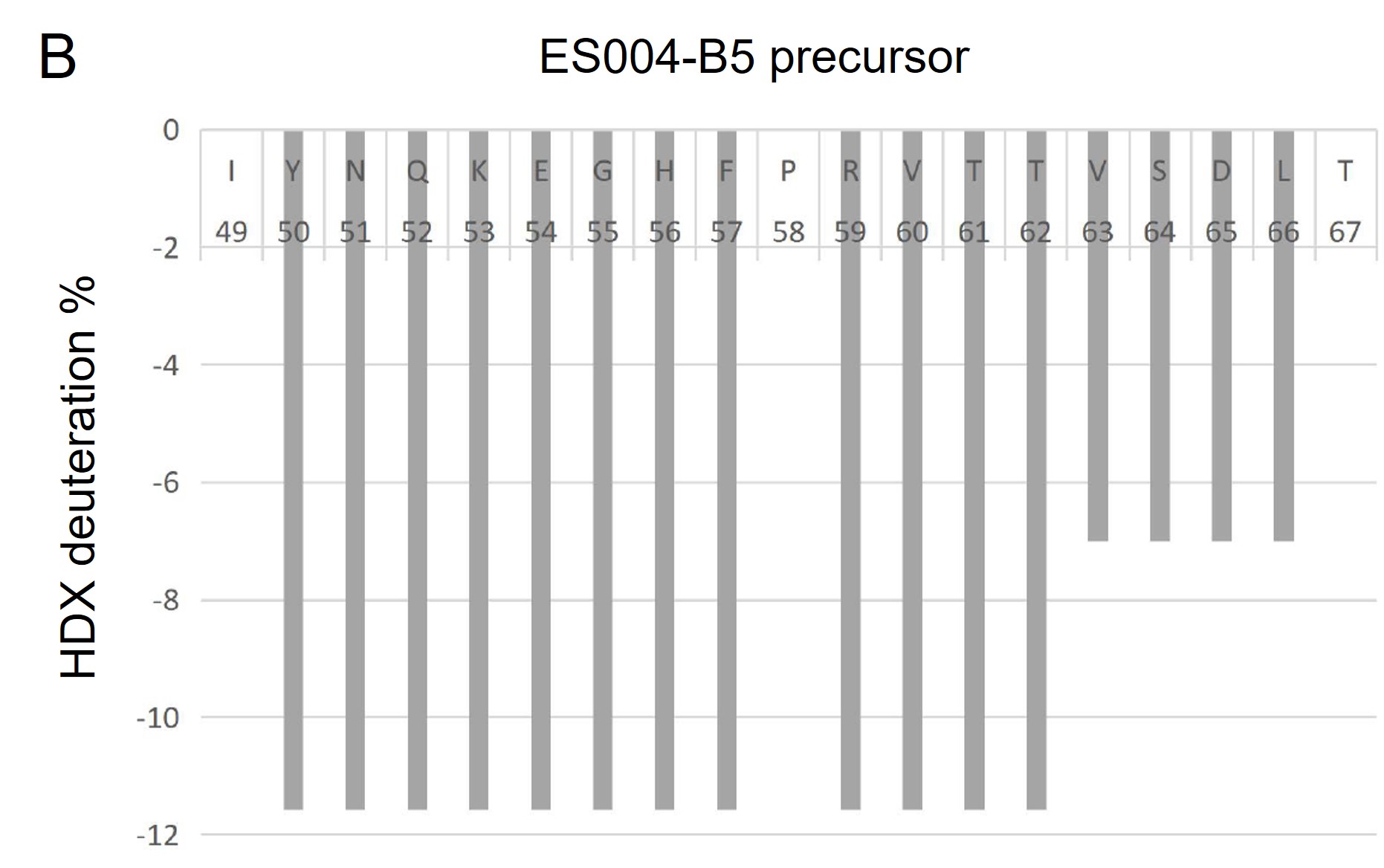

Supplement: Supplementary_material_tbae022 [file supplementary_material_tbae022.zip › Supplemental Figure 2B.jpg]

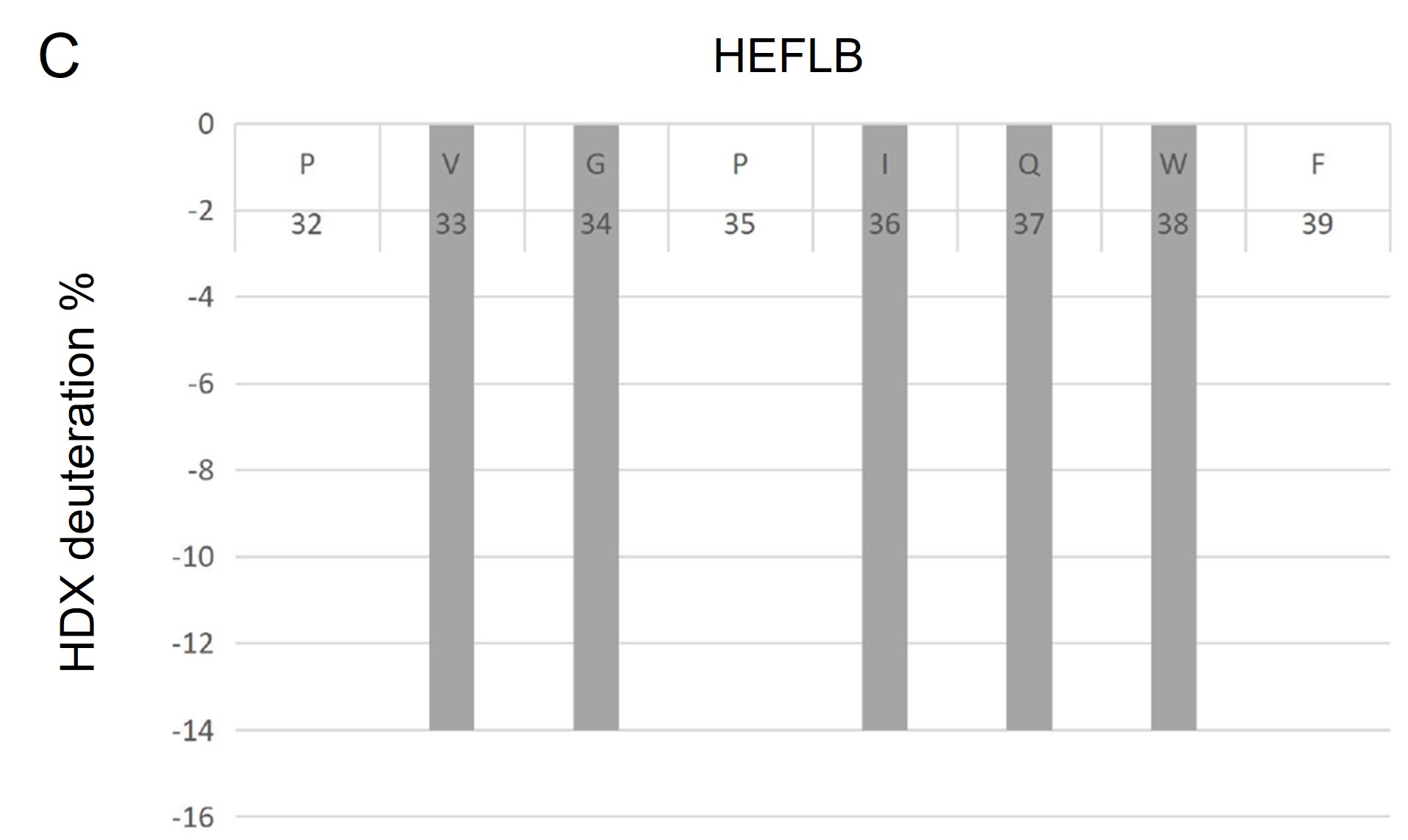

Supplement: Supplementary_material_tbae022 [file supplementary_material_tbae022.zip › Supplemental Figure 2C.jpg]

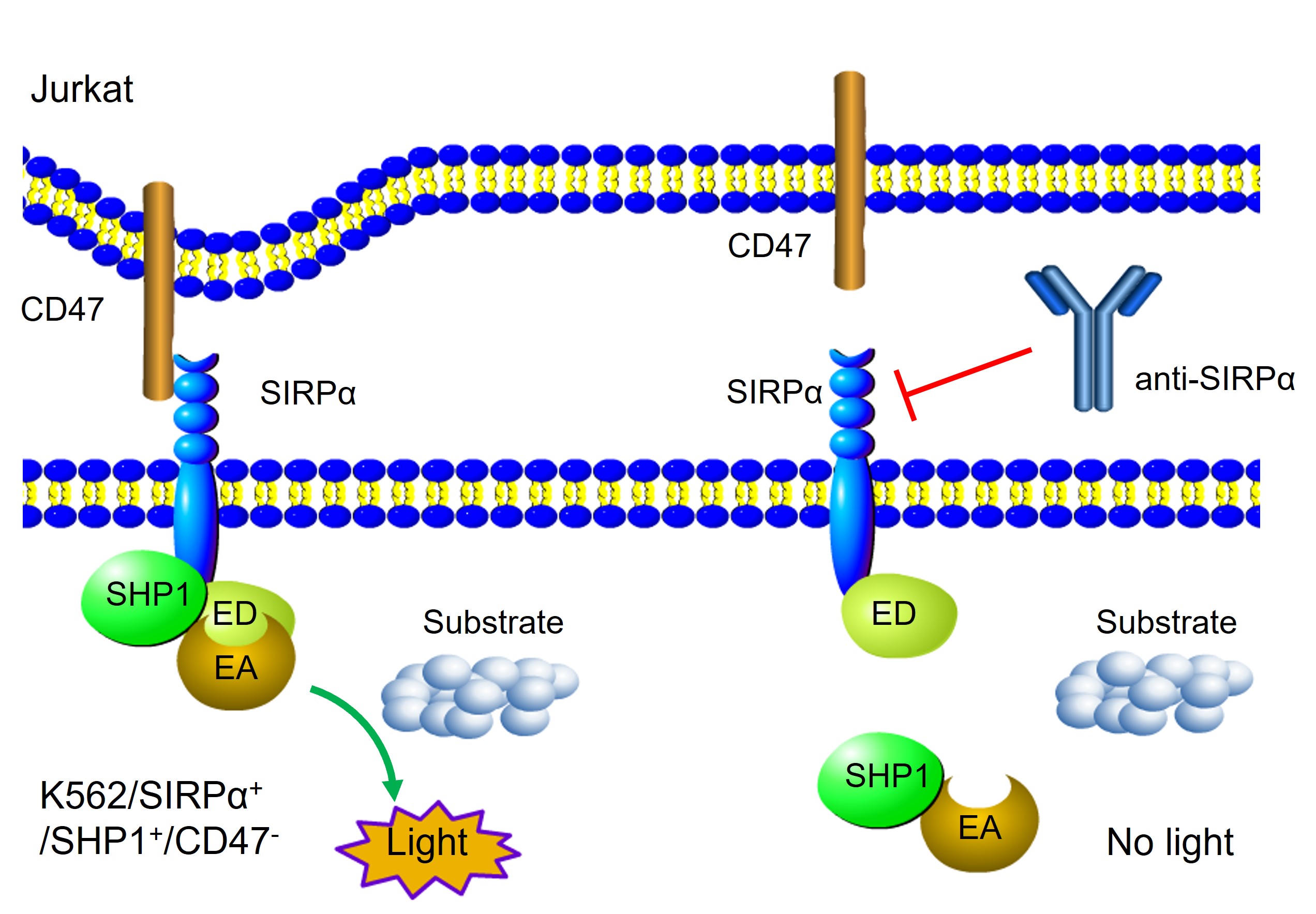

Supplement: Supplementary_material_tbae022 [file supplementary_material_tbae022.zip › Supplemental Figure 3.jpg]

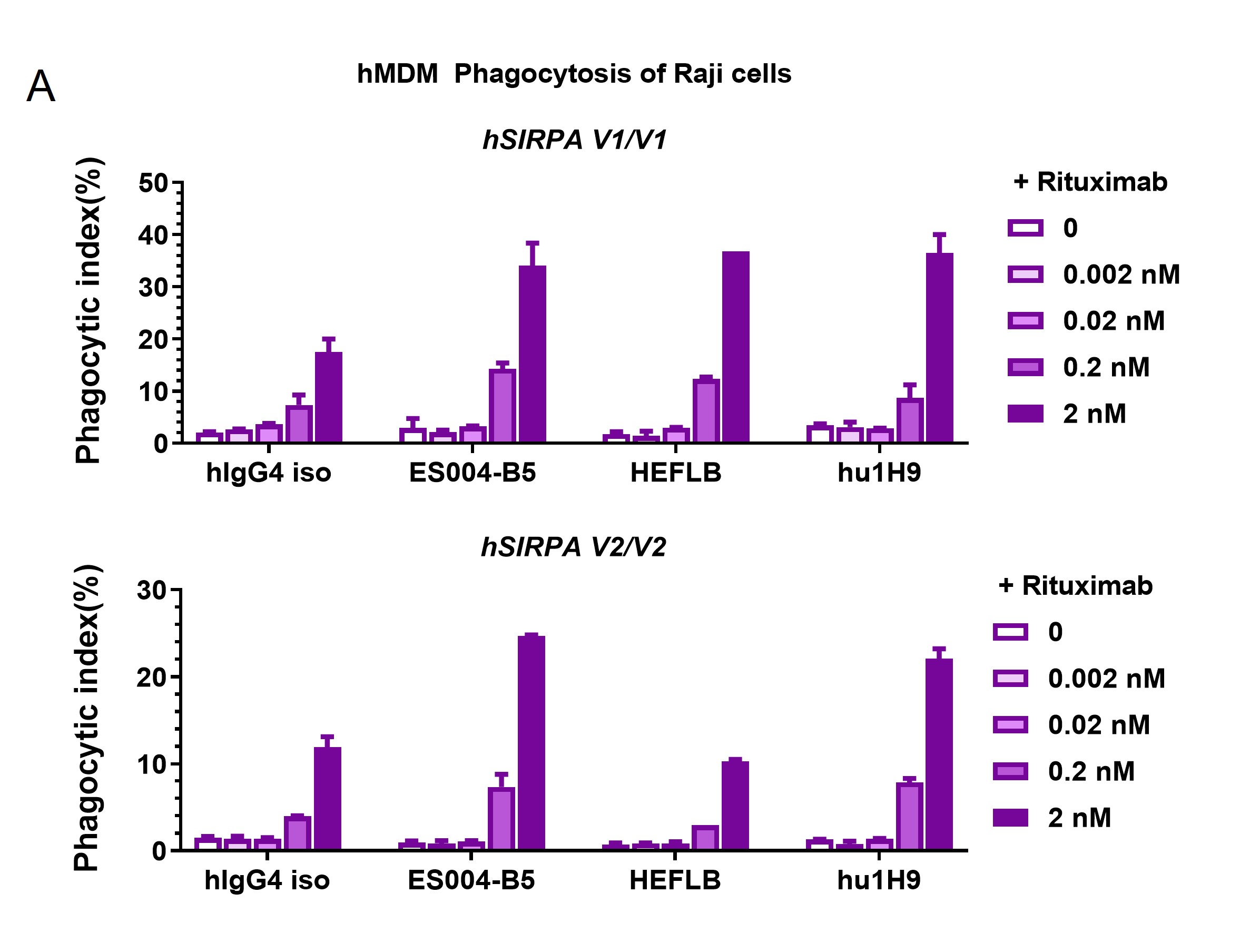

Supplement: Supplementary_material_tbae022 [file supplementary_material_tbae022.zip › Supplemental Figure 4A.jpg]

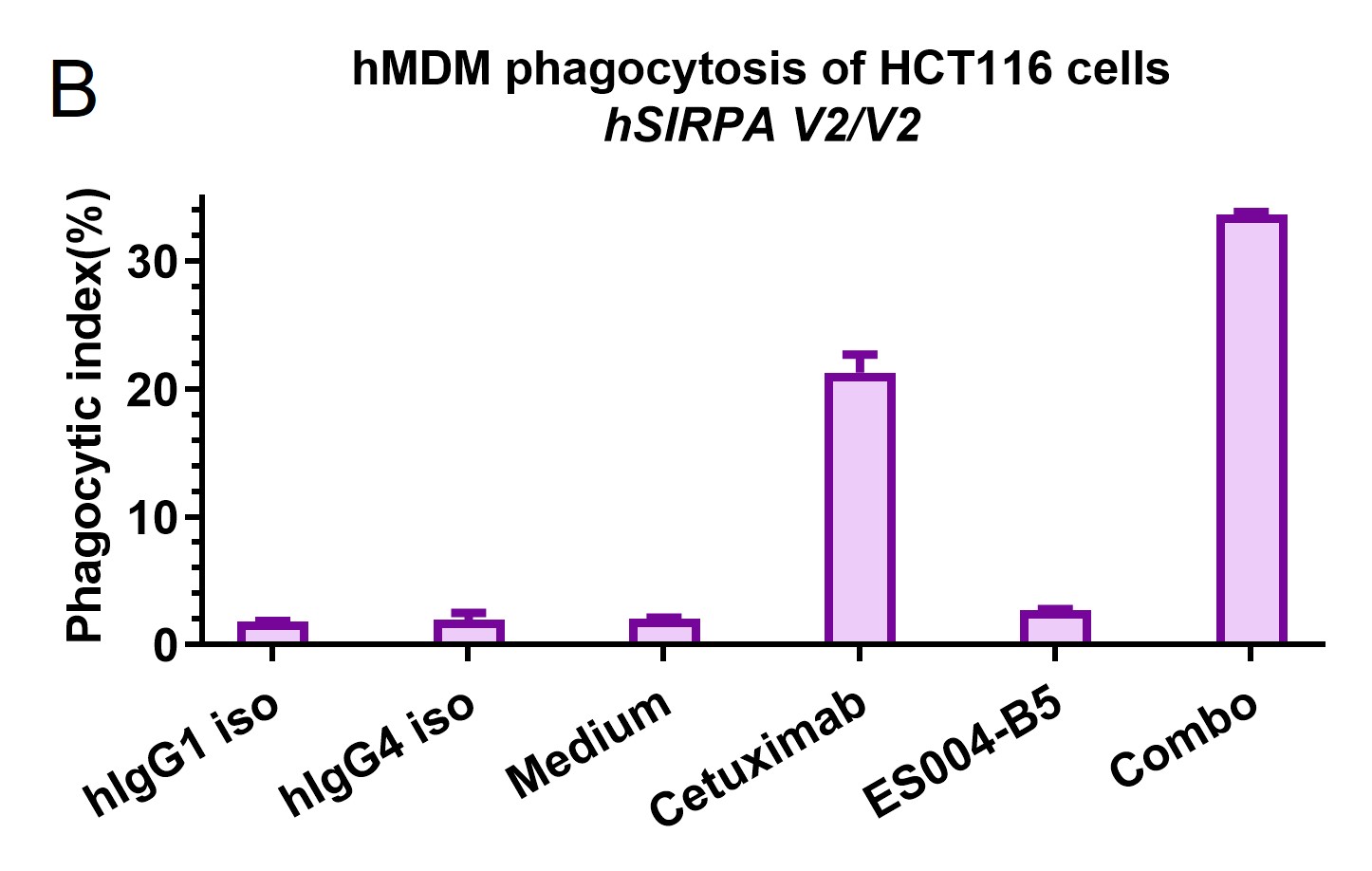

Supplement: Supplementary_material_tbae022 [file supplementary_material_tbae022.zip › Supplemental Figure 4B.jpg]

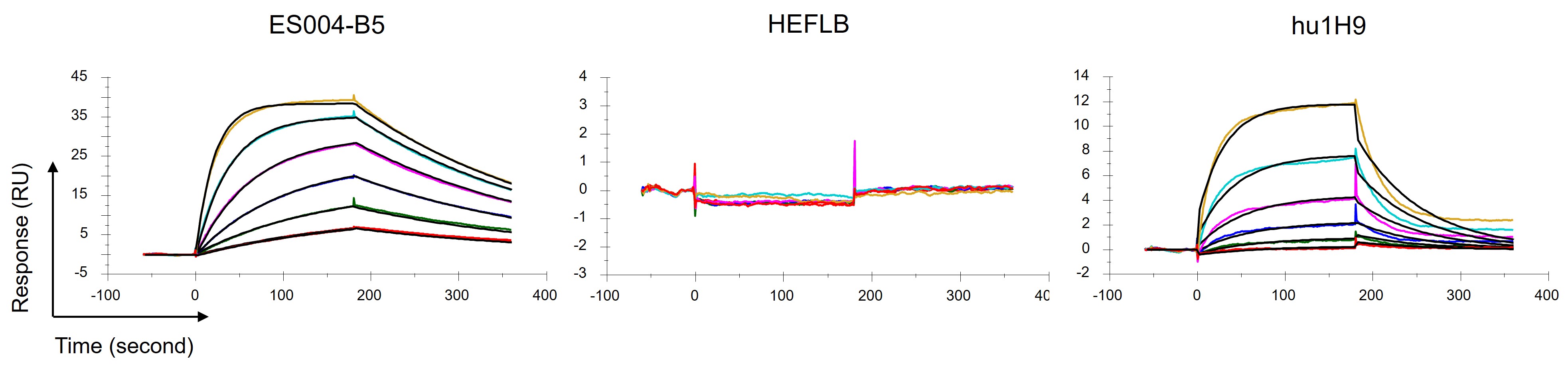

Supplement: Supplementary_material_tbae022 [file supplementary_material_tbae022.zip › Supplemental Figure 5.jpg]

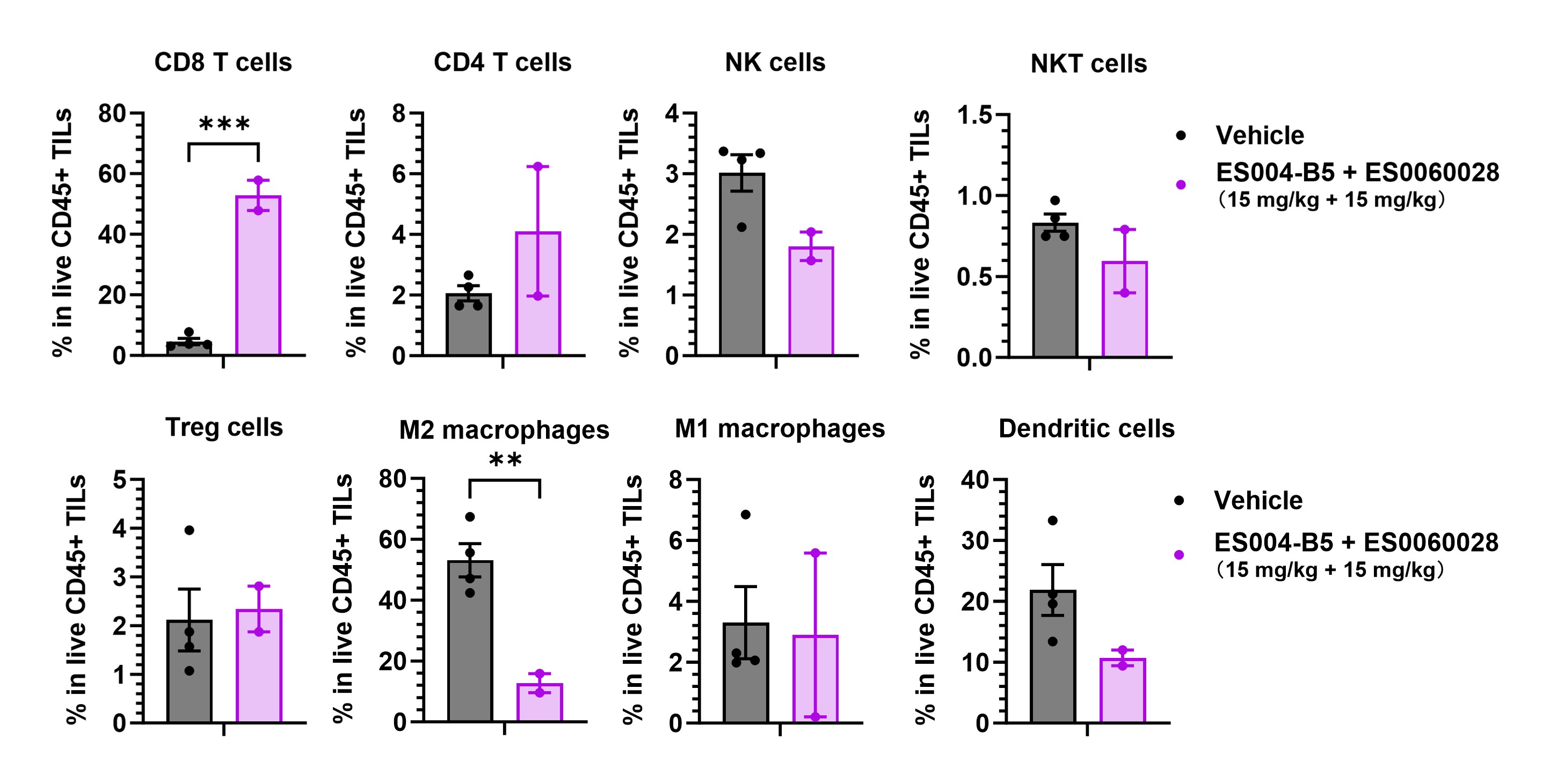

Supplement: Supplementary_material_tbae022 [file supplementary_material_tbae022.zip › Supplemental Figure 6.jpg]
